# The influence of electrochemical cycling protocols on capacity loss in nickel-rich lithium-ion batteries†

**DOI:** 10.1039/d1ta06324c

**Published:** 2021-10-11

**Authors:** Wesley M. Dose, Jędrzej K. Morzy, Amoghavarsha Mahadevegowda, Caterina Ducati, Clare P. Grey, Michael F. L. De Volder

**Affiliations:** Department of Engineering, University of Cambridge 17 Charles Babbage Road Cambridge CB3 0FS UK mfld2@cam.ac.uk; Department of Chemistry, University of Cambridge Lensfield Road Cambridge CB2 1EW UK cpg27@cam.ac.uk; Department of Materials Science and Metallurgy, University of Cambridge 27 Charles Babbage Road Cambridge CB3 0FS UK; The Faraday Institution, Quad One, Harwell Science and Innovation Campus Didcot OX11 0RA UK

## Abstract

The transition towards electric vehicles and more sustainable transportation is dependent on lithium-ion battery (LIB) performance. Ni-rich layered transition metal oxides, such as NMC811 (LiNi_0.8_Mn_0.1_Co_0.1_O_2_), are promising cathode candidates for LIBs due to their higher specific capacity and lower cost compared with lower Ni content materials. However, complex degradation mechanisms inhibit their use. In this work, tailored aging protocols are employed to decouple the effect of electrochemical stimuli on the degradation mechanisms in graphite/NMC811 full cells. Using these protocols, impedance measurements, and differential voltage analysis, the primary drivers for capacity fade and impedance rise are shown to be large state of charge changes combined with high upper cut-off voltage. Focused ion beam-scanning electron microscopy highlights that extensive microscale NMC particle cracking, caused by electrode manufacturing and calendering, is present prior to aging and not immediately detrimental to the gravimetric capacity and impedance. Scanning transmission electron microscopy electron energy loss spectroscopy reveals a correlation between impedance rise and the level of transition metal reduction at the surfaces of aged NMC811. The present study provides insight into the leading causes for LIB performance fading, and highlights the defining role played by the evolving properties of the cathode particle surface layer.

## Abstract 2

Tailored electrochemical protocols and characterisation methods provide a detailed account of degradation mechanisms in high energy lithium-ion batteries with nickel-rich cathodes.

## Graphical abstract

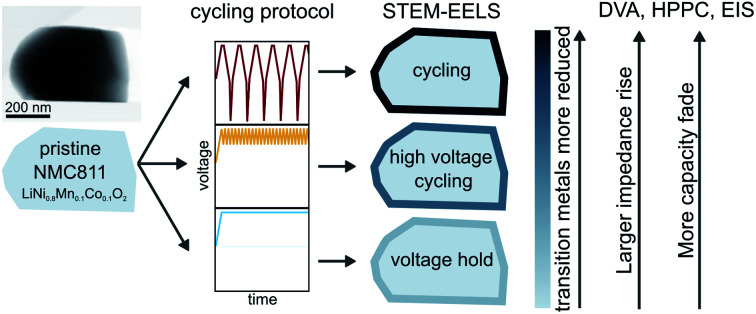

## 1. Introduction

With hybrid and electric vehicle (EV) sales and market shares continuously rising, their most prevalent way of reversibly storing energy – the lithium-ion battery (LIB) – has become increasingly important. LIBs with better performance are urgently needed, as higher specific capacity, rate capability, lifetime, safety, and environmental impact are key parameters for longer driving range, safer, and more sustainable EVs. Progress is limited by the cathode active material (CAM), as it tends to be the bottleneck of cost and performance.^1,2^ Among the CAMs, layered transition metal oxides, such as NMCs (LiNi_*x*_Mn_*y*_Co_1−*x*−*y*_O_2_) have been successfully applied commercially.^1^ In particular, high nickel content (Ni-rich) layered transition metal oxides such as NMC811 (LiNi_0.8_Mn_0.1_Co_0.1_O_2_) are especially attractive, as they provide larger specific capacities compared to lower nickel alternatives and have lower costs and resource issues due to reduced Co content. However, higher Ni content CAMs negatively impact the cell longevity.^3,4^

It has been suggested that the lattice collapse at high states of charge (SOC) is the main driver of capacity loss for many layered transition metal oxides.^5^ The repetitive lattice expansion and contraction is thought to lead to particle degradation in the form of inter- and intra-granular cracking.^4,6,7^ Oxygen release has been found to occur at similar high states of charge as the lattice collapse, leading to chemical oxidation of the electrolyte and transition metal dissolution.^8–11^ The oxygen release is also connected to surface reconstruction to spinel and rock salt-like phases (which are oxygen-deficient compared to layered NMC) that lead to hindrance of lithium (de)intercalation due to their lower ionic conductivity.^7,12^ A second suggested mechanism for impedance increase across the surface is a build-up of a resistive cathode electrolyte interphase (CEI) due to chemical (released, reactive oxygen) and electrochemical oxidation of the electrolyte.^13,14^ The surface reconstruction and CEI build-up are also more detrimental when paired with particle cracking and exposure of fresh surfaces to the electrolyte leading to additional oxygen release, reduction of new surfaces, and electrolyte oxidation.^4,15^

The abovementioned degradation processes have been reported for most NMC cathode compositions cycled under a wide range of conditions – *e.g.*, different upper cutoff voltage (UCV), C-rate, cycle number, *etc.* However, there is currently very limited understanding of the precise electrochemical cycling conditions that drive each degradation pathway. Instead, often a group of degradation processes, like surface reconstruction, CEI formation, Li/Ni site mixing, and particle cracking, are stated to be collectively responsible for the measured capacity fade and impedance rise.^16^ In this work, we study the specific origins of capacity fade and impedance rise in graphite/NMC811 cells. Targeted electrochemical aging protocols are used to link the applied electrochemical stimuli directly to the measured capacity loss and material degradation. Investigation of the full cell electrochemical data is coupled with differential voltage analysis (DVA), electrochemical impedance spectroscopy (EIS), focused ion beam – scanning electron microscope (FIB-SEM) tomography, and electron energy loss spectroscopy (EELS). The results from all these techniques are compared and contextualized in the Discussion section to provide new insights into the degradation pathways of LIBs with Ni-rich NMC cathodes.

## 2. Experimental

### 2.1 Electrodes

NCM811 cathodes and graphite anodes were prepared at large-scale in the Cell Analysis, Modeling, and Prototyping (CAMP) facility at Argonne National Laboratory. The cathodes consisted of 90 wt% NMC811 (Targray), 5 wt% polyvinylidene difluoride binder (PVDF; Solvay 5130), and 5 wt% conductive carbon (Timcal C45) cast onto 20 μm thick aluminium foil using *N*-methyl-2-pyrrolidone (NMP) as the solvent. The cathode sheets had loadings of 8.21 mg_NMC_ cm^−2^ corresponding to ∼1.52 mA h cm^−2^ based on 185 mA h g_NMC_^−1^. The anodes consisted of 91.83 wt% artificial graphite (Hitachi MagE3), 2 wt% conductive carbon (Timcal C45), 6 wt% PVDF binder (Kureha 9300), and 0.17 wt% oxalic acid cast onto 10 μm thick copper foil using NMP as the solvent. The anode sheets had loadings of 5.83 mg_Gr_ cm^−2^ corresponding to ∼1.92 mA h cm^−2^ based on 330 mA h g_Gr_^−1^. After drying electrodes were calendered using a heated (80 °C) two-roller hydraulic-driven roll press (A-PRO Co.) to 30% porosity. Circular electrodes with 14 mm (cathode) and 15 mm (anode) diameters were punched and dried at 120 °C for at least 12 h under dynamic vacuum before being transferred to an argon filled glove box (<0.5 ppm O_2_ and H_2_O; MBraun). The electrode capacity balancing of anode and cathode (N : P ratio) was set to ≈1.20 : 1.00 with a cell upper cutoff voltage (UCV) of 4.2 V.

### 2.2 Cell assembly

2032-type coin cells (Hohsen) were assembled in a full cell setup with a 14 mm diameter cathode, 15 mm diameter anode, and 16 mm diameter polymer separator (MTI; dried for at least 12 h under dynamic vacuum at 60 °C) soaked in 40 μL of LP57 electrolyte (SoulBrain, 1 M LiPF_6_ in ethylene carbonate (EC) : ethyl methyl carbonate (EMC) 3 : 7 v/v).

### 2.3 Cycling protocols

**Fig. 1:**
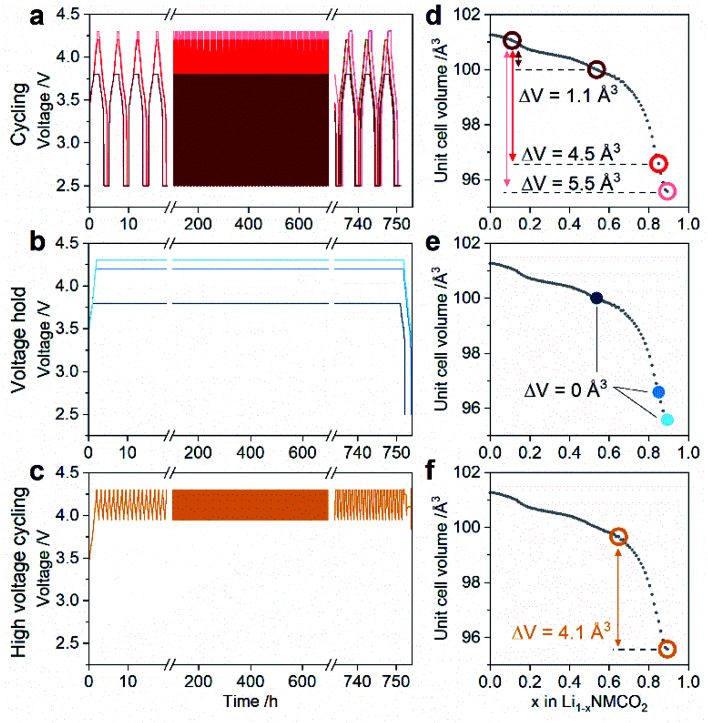
Electrochemical protocols applied to graphite/NMC811 full cells. (a–c) Electrochemical protocols and (d–f) the corresponding change in NMC811 unit cell volume during aging for cells undergoing (a and d) constant current–constant voltage cycling, (b and e) voltage hold, and (c and f) high voltage cycling. The first and last 20 h of the 750 h duration aging protocols are enlarged in (a–c), and formation and diagnostic cycles are omitted for clarity. In (d–f) the unit cell volume data (gray data points) for NMC811 during charge to 4.4 V *vs.* Li/Li^+^ is reproduced from Fig. 2 in ref. 18. The colored data points indicate the NMC811 unit cell volume change based on the voltage range in each of the aging protocols.

All full cells were initially formed by three cycles at C/20 (assuming a practical capacity of 185 mA h g_NMC_^−1^) between 2.5–3.8 V, 2.5–4.2 V, or 2.5–4.3 V. Afterwards, the cells were aged by one of three electrochemical aging protocols, classified as cycling (CYC), voltage hold (VH), and high voltage cycling (HVC). Cells were measured in duplicate or triplicate for each condition. The aging time in each protocol was kept constant at 750 h (∼31 days). In the CYC protocol (Fig. 1a), full cells were cycled at C/2 using a constant current–constant voltage (CCCV) charge and CCCV discharge between 2.5–3.8 V, 2.5–4.2 V, or 2.5–4.3 V (Biologic BCS 805 series). The formation and aging UCV for a given cell were kept the same. The number of cycles was constrained to 150 cycles by using a fixed time of 2.5 h for charge and discharge (*i.e.* 5 h per cycle). The VH protocol (Fig. 1b) involved charging the full cell at C/2 to 3.8, 4.2, or 4.3 V – equivalent to the formation UCV – followed by a 750 h voltage hold, after which the cell was discharged at C/2 to 2.5 V (Arbin BT2043). In the HVC protocol (Fig. 1c), the cell was charged at C/2 to 4.3 V and subsequently cycled with the same applied current between 3.95–4.3 V until 750 h had elapsed, after which the cell was discharged at C/2 to 2.5 V (Arbin BT2043). After aging, all cells were subjected to three diagnostic cycles at C/20 between 2.5–3.8 V, 2.5–4.2 V, or 2.5–4.3 V, with the UCV for each cell equivalent to that during formation and aging.

The full cell impedance was measured after the formation cycles and after aging by a hybrid pulse power characterization (HPPC) test protocol.^61^ In preparation for the HPPC test the cells were charged at C/2 to the UCV (3.8, 4.2, or 4.3 V). In this work, the HPPC pulse sequence encompasses a 10 s 3C discharge current pulse and a 10 s 2.25C charge pulse separated by 40 s at open circuit voltage (OCV). Prior to the pulse sequence, the cells were allowed to rest at OCV for 1 h. To obtain the potential-dependent impedance, this pulse sequence was repeated at 10% depth of discharge (DOD) intervals, with the intermediate discharge performed using constant current (CC) at C/2. Note that the 10% DOD segments between the pulses are not adjusted to reflect the capacity of the aged cell; and that during the HPPC pulse sequence upper and lower cutoff voltages (2.5 V and 4.4 V, respectively) are set to prevent overcharge or overdischarge of the cell. ASI values are calculated from the change in cell voltage during the current pulses, using the geometric area of the cathode (1.54 cm^2^) for the calculation. For cells aged by the CYC protocol, the full cell impedance was also measured after the formation cycles and after the aging and diagnostic cycles by electrochemical impedance spectroscopy (EIS). Potential-controlled EIS was conducted in a frequency range of 500 kHz to 10 mHz with an AC voltage perturbation of 5 mV (Biologic VMP3). Before the measurement, the full cells were charged at C/20 to 3.8 V and held at 3.8 V for 10 h after which the current is <C/5000. All electrochemical protocols were performed in climate chambers set at 25 °C. Two or three cells were evaluated for each condition to ensure reproducibility, which is indicated by error bars in the respective figures.

### 2.4 Electrochemical examination of harvested cathodes

At the end of the full cell electrochemical protocol described above, the coin cells were brought to the discharged state at a slow rate (2.5 V; C/20) and then disassembled in an Ar filled glove box. The aged NMC811 electrodes were harvested and, without rinsing, immediately assembled into half-cells with a lithium metal counter electrode (MTI) and a fresh separator soaked in 40 μL of fresh LP57 electrolyte. Half-cells with pristine NMC811 cathodes – *i.e.* not cycled in a full cell – were also built. NMC811 half-cells were cycled 5 times at C/20 between 2.5–4.5 V *vs.* Li/Li^+^.

### 2.5 Electron microscopy

To prepare samples for STEM-EELS experiments, cathodes were harvested from cycled coin cells, washed with diethyl carbonate, dried in an evacuated antechamber for 30 minutes and left to fully dry overnight in the Ar filled glovebox. Then, the cathodes were scraped with a disposable spatula and resulting powder was dusted onto a copper, holey carbon TEM grid. Samples were transferred in an Ar filled, sealed bag which was opened just before mounting the sample and inserting the holder into the microscope (exposed to air for about 1 minute). A FEI Tecnai Osiris transmission electron microscope with a high-brightness X-FEG electron gun operated at 200 kV accelerating voltage was used for electron energy loss spectroscopy in scanning mode. The spectra were collected using a Gatan Enfinium ER 977 spectrometer. Dual-EELS mode was used at 0.25 eV per channel dispersion to collect the low- (including the zero-loss peak) and core-loss spectra simultaneously at each probe position, forming a spectrum image. Pixel size of about 1 nm and 50 ms dwell time were typically used. The energy resolution was determined as the full width at half maximum of the zero-loss peak and was about 1 eV. Resulting spectrum images were analyzed using an open-source Python package HyperSpy.^62^ Details on data processing procedures can be found in the ESI Note S3.†

Zeiss CrossBeam 540 dual beam FIB-SEM microscope was used to perform FIB-SEM tomography. A 1 μm thick Pt-based protective layer was deposited on the top surface of a harvested and washed cathode. Then, alignment marks were milled in the Pt layer and covered with a 1 μm carbon layer. Initial FIB rough milling to prepare a 20 × 30 × 30 μm volume was made using 30 kV Ga-ion beam at 30 nA beam current. Then, the milling of tomographic slices of about 50 nm in nominal thickness was done using 30 kV 1.5 nA Ga ion beam. After each slice, a backscattered electron image was acquired using 2.6 kV accelerating voltage, 12 nA beam current at about 25 nm pixel size.

## 3. Results

### 3.1 Electrochemical protocols

Fig. 1a–c shows the three types of electrochemical aging protocols applied to graphite/NMC811 full cells in this work. These are classified as CYCling (CYC), Voltage Hold (VH), and High Voltage Cycling (HVC). Full details of the conditions for each protocol are given in the Experimental section. First, the CYC protocol (Fig. 1a) assesses the degradation during ‘standard’ cycling with variable UCV – mid (3.8 V) or high (4.2 and 4.3 V). Second, the VH protocols (Fig. 1b) evaluate the effect of a sustained voltage hold at mid- (3.8 V) or high-voltage (4.2 and 4.3 V). Finally, in the HVC protocol (Fig. 1c), the cell is repeatedly cycled between 3.95–4.3 V, and is designed to test the stability of the full cell when subjected to sustained high voltage combined with a SOC fluctuation. In this voltage window, the NMC811 cathode shows a peak in the d*Q*/d*V* at 4.20 V *vs.* Li/Li^+^ on charge and discharge (Fig. S1†). Similar electrochemical features in LiNiO_2_ are linked to a phase transition from H2 to H3,^17^ although the two-phase behavior is not observed for NMC811.^18^ To compare and contrast the effect of the electrochemical stimuli on the cell degradation, the aging time is kept constant at 750 h (∼31 days).

An important point of difference in each of the protocols, and a key motivation in their design, is the change in the NMC811 cathode state of charge (ΔSOC) and unit cell volume during aging. The latter is shown in Fig. 1d–f. For the VH protocol the ΔSOC and the unit cell volume change are nominally zero since fixing the cell voltage also fixes the SOC of the NMC cathode. For CYC and HVC the ΔSOC depends on the size of the voltage window, while the unit cell volume change depends on both the voltage window and the rate of change of the unit cell volume, which is SOC dependent. CYC between 2.5–3.8 V yields a ΔSOC of 43% and a small volume change of 1.1 Å^3^ due to the slow rate of volume change for SOC <60%. Note that ΔSOC is calculated from the C/2 discharge capacity in the third aging cycle and a theoretical capacity for NMC811 of 275.5 mA h g^−1^. Increasing the voltage window from 2.5–4.2 V to 2.5–4.3 V, an increase of only 100 mV, affects the ΔSOC minimally (71 and 73%, respectively) but the volume change increases significantly from 4.5 Å^3^ to 5.5 Å^3^ in response to the rapidly contracting unit cell at high SOC. The latter is capitalized upon in the HVC protocol, with the narrow 350 mV window (3.95–4.3 V, compared to 1700 and 1800 mV for CYC 2.5–4.2 V and 2.5–4.3 V, respectively) giving a modest 17% ΔSOC but a significant volume change of 4.1 Å^3^. From a particle degradation perspective, this condition is expected to be the harshest, combining large anisotropic volume change with a large number of cycles (>800) across the aging time. In the following sections, we explore the effect of these protocols on the capacity loss and material degradation.

### 3.2 Full cell capacity loss

**Fig. 2:**
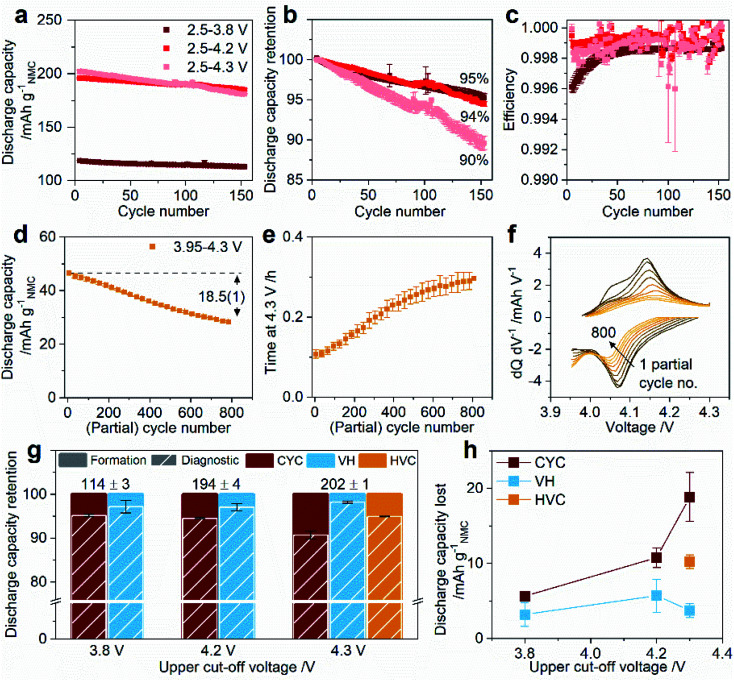
Full cell graphite/NMC811 performance during aging and diagnostic cycles. For aging by cycling (CYC): (a) discharge capacity, (b) capacity retention, and (c) coulombic efficiency for cycling between lower cut-off 2.5 V and upper cut-off 3.8 V, 4.2 V, and 4.3 V. For aging by high voltage cycling (HVC): (d) discharge capacity, (e) time on the 4.3 V constant voltage charge step, and (f) representative differential capacity *versus* voltage. For the diagnostic cycles: (g) discharge capacity retention, and (h) discharge capacity lost as a function of upper cut-off voltage after aging by CYC, VH, and HVC. In (a), (d), and (h) capacity is normalized to the NCM811 active material mass. Discharge capacity retention in (b) is normalized to the third C/2 aging cycle. In (f) every 100^th^ partial cycle is presented. Capacity retention and capacity lost in (g) and (h) are measured between the third C/20 formation cycle and after aging in the third C/20 diagnostic cycle. In (g) the values quoted at the top of the columns are the average discharge capacity (in mA h g_NMC_^−1^) in the third C/20 formation cycle for each upper cut-off voltage, with the error representing the spread of 4–7 duplicate cells. In (h) the lines connecting the data points are to guide the eye only. Error bars in all plots represent the spread in the data for two duplicate cells.

The discharge capacity, capacity retention, and coulombic efficiency during the C/2 aging cycles for the CYC protocol are shown in Fig. 2a–c. Voltage profiles and differential capacity plots for select cycles during CYC aging are provided in Fig. S2.† Increasing the UCV from 3.8 to 4.2 V increases the capacity from 118.5(1) to 195.9(8) mA h g_NMC_^−1^, but has little effect on the capacity retention over 150 cycles (95.3(5) and 94.5(3)%, respectively). However, further increasing the UCV to 4.3 V results in noticeably poorer capacity retention (89.6(8)%) with only a modest (initial) capacity gain, delivering 6(2) mA h g_NMC_^−1^ more than with a 4.2 V UCV.

The discharge capacity during HVC aging is much lower due to the narrow 3.95–4.3 V window, initially delivering 47.0(9) mA h g_NMC_^−1^ (Fig. 2d). Over the 750 h aging period the HVC cells complete >800 partial cycles and the capacity decreases by 18.5(1) mA h g_NMC_^−1^ (the data for each duplicate cell is shown in Fig. S3†). However, as will be seen later, not all this capacity is truly “lost” since a significant portion of it is recovered when the C-rate is slowed to C/20 and the voltage window extended to 2.5–4.3 V. The time spent on the 4.3 V CV step in each partial cycle increases almost three-fold during HVC aging (Fig. 2e), indicating rising kinetic and/or potential hindrance for lithium extraction, which correlates with the increasing over-potential for the 4.14 V charge process and the corresponding 4.07 V discharge process, as shown in the differential capacity plot in Fig. 2f.

Before and after the aging cycles the cells are cycled three times at C/20 between 2.5 V and the UCV employed in the aging protocol, *i.e.* 3.8, 4.2, or 4.3 V. These are referred to as the formation and diagnostic cycles, respectively. Fig. 2g and h show the discharge capacity retention and capacity lost between the formation and diagnostic cycles for all protocols as a function of UCV – representative full cell voltage profiles and differential voltage plots for these cycles are shown in Fig. S4.† Of the three types of protocols, and irrespective of UCV, aging by VH causes the least capacity loss, indicating that time spent at mid- and high-voltage (without SOC change) is not a major contributor to capacity fade in these cells. The CYC protocol led to greater capacity loss than HVC despite the much smaller number of cycles, with a strong dependence on the UCV. Capacity retention for a 3.8 and 4.2 V UCV are similar, 95.2(3) and 94.5(2)%, consistent with the result in the C/2 aging cycles; however, as Fig. 2h shows, twice as much capacity is lost with a 4.2 V UCV (11(1) mA h g_NMC_^−1^ compared to 5.6(2) mA h g_NMC_^−1^ for a 3.8 V UCV). CYC with a 4.3 V UCV is the protocol most detrimental to capacity loss retaining only 90.7(9)% of the original 202(1) mA h g_NMC_^−1^ capacity after the 750 h aging time. The capacity retention and capacity lost after aging by the HVC protocol are 94.9(1)% and 10.3(9) mA h g_NMC_^−1^, respectively, both improved compared to aging by the CYC protocol with the same 4.3 V UCV. This is despite completing >800 partial cycles compared to 150 cycles in the CYC protocol. While the cycle number is vastly different, the total capacity delivered over the aging period for the two protocols is similar; 28.8(1) A h g_NMC_^−1^ for CYC 2.5–4.3 V compared to 29.7(7) A h g_NMC_^−1^ for HVC 3.95–4.3 V. Therefore, the poorer capacity retention and larger capacity loss for the CYC 2.5–4.3 V protocol indicates that fewer repetitions over the full SOC range are more detrimental than a much larger number of cycles in a narrower voltage range, even with an equally high 4.3 V UCV and a higher fraction of the total time spent at higher voltages.

### 3.3 Capacity loss mechanisms

**Fig. 3:**
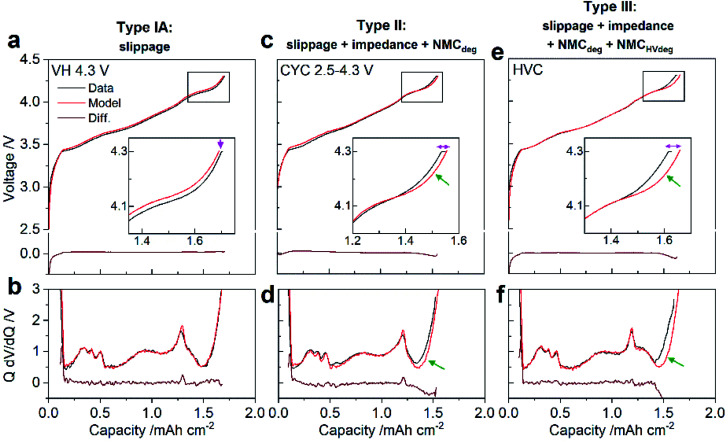
Identification of capacity loss mechanisms using differential voltage analysis (DVA). (a, c and e) Charge voltage profiles and (b, d and f) differential voltage *versus* capacity for the third C/20 diagnostic cycle (black) together with the modelled profiles from DVA (red), and the difference between model and data (brown). Examples of cycling conditions giving rise to degradation classified as: (a and b) type IA (*e.g.* voltage hold [VH] at 4.3 V), (c and d) type II (*e.g.* cycling [CYC] between 2.5–4.3 V), and (e and f) type III (*e.g.* high voltage cycling [HVC]) are shown. The insets in (a, c and e) show a magnified view of the voltage profiles nearing the end of charge. Magenta arrows highlight the closely modelled constant-current charge capacity (down arrow) or the capacity difference between the data and model (horizontal magenta arrow). Green arrows point to regions where the model deviates from the data. The derivative, (d*V*/d*Q*), in (b, d and f) is multiplied by the cell capacity (*Q*) in the given cycle, which normalizes the derivative based on the cell capacity.

To deconvolute the capacity fade mechanisms taking place in each protocol, differential voltage analysis (DVA) is applied to the C/20 formation and diagnostic cycles. These low C-rate cycles were selected to mitigate the effects of impedance on the analysis. DVA involves fitting differential voltage (d*V*/d*Q*) half-cell data (collected for the cathode and anode separately) to the full cell data, while refining the relative alignment and overall capacity of the half-cell curves to minimize the difference between the model and the data,^19–21^ as illustrated in our prior work on similar graphite/NMC811 full cells.^22^ The results from the fitting quantify the capacity loss attributable to degradation at the cathode, anode, and from electrode slippage, the latter being caused by side reactions at either electrode. More details on DVA theory and the fitting performed in this work can be found in ESI Note S1, Fig. S5 and Table S1.† Note that only the CC portion of the CCCV charge can be modelled by DVA since in the CV step d*V*/d*Q* = 0. Also, note that cycling conditions with a 3.8 V UCV could not be modelled since DVA requires features from a wider SOC range to obtain a reliable fitting result. Based on the DVA, all remaining cycling conditions tested could be classified according to modes of degradation referred to here as type I (A or B), II, or III, with the number of active degradation modes increasing from type I to type III. In type I, capacity loss is due to electrode slippage (type IA) or electrode slippage and NMC cathode degradation (NMC_deg_) (type IB). Capacity loss from electrode slippage is due to the loss of cyclable lithium caused by the lithium trapped in the solid electrolyte interphase (SEI) on the graphite negative electrode,^21,23,24^ and its continued formation and repair during cycling increasing slippage, while NMC_deg_ is the fitted capacity lost attributable to degradation of the NMC cathode, *i.e.*, the lithium that can be extracted from the cathode, as determined from DVA. In type II, in additional to capacity loss from slippage and NMC_deg_, capacity is also lost due to an increased cell impedance. Finally, in type III an additional NMC cathode degradation mode is active, for which capacity loss occurs specifically at NMC potentials >4.1 V *vs.* Li/Li^+^ and is thus termed NMC high voltage degradation (NMC_HVdeg_). Examples of cycling conditions giving rise to degradation by type I, II, and III are shown in Fig. 3, illustrated by the fitted voltage profiles and capacity normalized d*V*/d*Q* plots for the charge of the third C/20 diagnostic cycle. Table 1 quantifies the amount of capacity loss attributed to each degradation mode, and compares the total modelled capacity lost to the measured capacity lost. Data for the conditions not shown here are provided in Fig. S6 and Table S2† – the latter summarizes all analyzed conditions. As this analysis is necessarily conducted on the diagnostic C/20 cycles, only the degradation processes that result in capacity loss at this C-rate are detected by DVA. Capacity loss below a 1 mA h g_NMC_^−1^ threshold was deemed insignificant. It is interesting to note that graphite anode degradation (as determined by DVA, which is distinct from electrode slippage due to SEI formation) is generally <0.8 mA h g_NMC_^−1^ (Table S1†) suggesting little, or no degradation of the graphite under the cycling conditions employed in this work.

**Table 1:** Breakdown of the capacity loss mechanisms determined by differential voltage analysis (DVA). Modelled and measured capacity lost for the examples highlighted in Fig. 3 are given. The capacity lost is measured between the third C/20 formation cycle and after aging in the third C/20 diagnostic cycle

	Type IA: slippage		Type II: slippage + impedance + NMC_deg_		Type III: slippage + impedance + NMC_deg_ + NMC_HVdeg_	
Aging condition	VH 4.3 V		CYC 2.5–4.3 V		HVC	
Degradation mode	Capacity lost (mA h g_NMC_^−1^)	Est.% overall	Capacity lost (mA h g_NMC_^−1^)	Est.% overall	Capacity lost (mA h g_NMC_^−1^)	Est.% overall
Slippage^a^	4(1)	90	17.6(3)	77	2(2)	18
Impedance (C/20)^b^	0.3	5	1.7	7	1.2	11
NMC_deg_^a^	0(2)	5	4(1)	16	3(3)	23
NMC_HVdeg_^c^	—	—	—	—	5(1)	48
Total modelled	5(3)	100	24(1)	100	11(6)	100
Measured^d^	4.6		23.2		11.0	

a Capacity from electrode slippage and NMC_deg_ were determined from DVA. NMC_deg_ is the fitted capacity lost attributable to degradation of the NMC cathode.

b Capacity lost due to impedance is taken as the difference in the CV charge capacity for the formation and diagnostic cycles, which both have a C/40 cutoff current.

c NMC_HVdeg_ is the NMC cathode capacity loss that occurs specifically at NMC potentials >4.1 V *vs.* Li/Li^+^, and is in addition to NMC_deg_ which is lost evenly across the entire SOC. More details on how NMC_HVdeg_ is calculated are provided in ESI Note S2.

d Measured capacity loss is the difference in the charge capacity at the end of the CC segment for the formation and diagnostic cycles, at a C/20 rate.

Cycling condition VH 4.2 V (Fig. S6a and b†) and VH 4.3 V (Fig. 3a and b) are classified as type IA, and CYC 2.5–4.2 V (Fig. S6c and d†) as type IB. For type I, the measured voltage and d*V*/d*Q* profiles are well fitted across the entire SOC, and the measured CC charge capacity is accurately predicted (insets in Fig. 3a, S6a and c†). In Fig. 3c and d the modelled voltage and d*V*/d*Q* profiles for CYC 2.5–4.3 V deviate from the data at high SOC (green arrows), and while the modelled CC capacity does not match with the measured CC capacity, it agrees well with the measured CCCV capacity (inset in Fig. 3c). This indicates that in addition to slippage and NMC_deg_, capacity is also lost due to an increased cell impedance; CYC 2.5–4.3 V is therefore classified as type II. Although HVC shows less capacity loss than CYC 2.5–4.3 V, it has more degradation modes and is classified as type III. Like CYC 2.5–4.3 V, the modelled voltage and differential voltage profiles for HVC (Fig. 3e and f) deviate from the data at high SOC (green arrows); in contrast, however, the model vastly overestimates both the measured CC and CCCV capacity (inset in Fig. 3e). It is unlikely that the cell impedance is responsible for this difference since the measured impedance for HVC is less than that for CYC 2.5–4.3 V (see below). Since the NMC cathode dominates the high SOC portion of the full cell voltage and d*V*/d*Q* profiles (see Fig. S5†), the mismatch observed suggests a second NMC capacity loss process is active. Unlike NMC_deg_ which models capacity lost evenly across the entire SOC, the additional NMC capacity loss occurs specifically at NMC potentials >4.1 V *vs.* Li/Li^+^. It is thus termed NMC high voltage degradation (NMC_HVdeg_). NMC_HVdeg_ is not incorporated into the current DVA model and therefore details on how NMC_HVdeg_ is quantified in this work are provided in the ESI Note S2 and in Fig. S7.† After this analysis, in all cases the sum of the modeled capacity loss agrees well with the measured capacity loss, as shown in Tables 1 and S2.†

### 3.4 Cell impedance rise

**Fig. 4:**
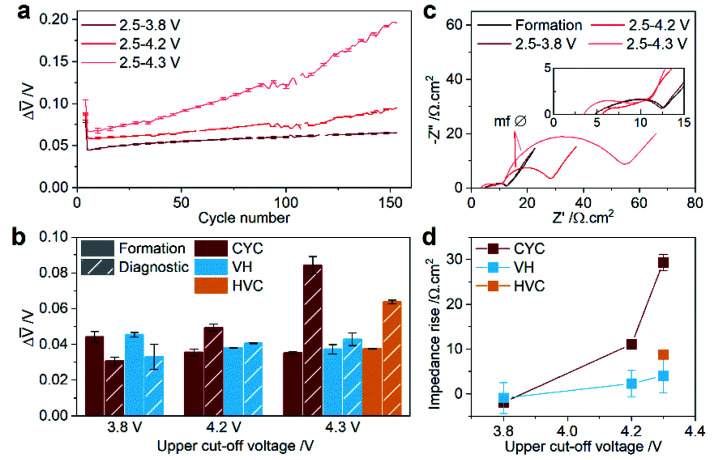
Full cell impedance rise before, during, and after aging by constant current–constant voltage cycling (CYC), voltage hold (VH), and high voltage cycling (HVC). (a) Mean voltage difference between charge and discharge (Δ*V̄*) during aging at C/2 by CYC. (b) Δ*V̄* in the third formation cycle and after aging in the third diagnostic cycle as a function of upper cut-off voltage. (c) AC impedance Nyquist plot for cells before and after aging by CYC. Spectra are measured at a full cell voltage of 3.8 V and at 25 °C. One representative spectrum is shown for a cell after formation (3 cycles between 2.5–4.3 V) for comparison. Inset shows a zoomed-in plot of the high frequency region. (d) Area specific impedance rise at ∼3.7 V full cell voltage determined in hybrid pulse power characterization (HPPC) measurements as a function of upper cut-off voltage. Error bars on the plots represent the spread of two or three duplicate cells.

The role of the electrochemical stimuli during aging on the cell impedance is explored by three approaches in Fig. 4. First, the evolving cell polarization during CYC aging at C/2 is tracked by the difference in the charge-averaged mean voltage between charge and discharge (Δ*V̄*, Fig. 4a). The initial Δ*V̄* is larger for higher UCV, and thereafter shows clear UCV dependent behavior. For CYC 2.5–3.8 V, Δ*V̄* remains fairly constant over the 150 cycles, while for CYC 2.5–4.2 V it shows only a slight increase toward the end of aging. Conversely, CYC 2.5–4.3 V exhibits a rapidly increasing Δ*V̄* across the aging period. Fig. 4b compares Δ*V̄* in the third C/20 formation and diagnostic cycle for the different aging protocols as a function of UCV. With a 3.8 V UCV and for VH aging, the cell polarization at C/20 decreases and stays the same, respectively. A rise in cell polarization, in increasing order, is noted for CYC 2.5–4.2 V, HVC, and CYC 2.5–4.3 V. For cells aged by CYC, the AC impedance was determined by electrochemical impedance spectroscopy (EIS) in two-electrode graphite/NMC811 full cells before and after aging. Three features are evident in the Nyquist plot in Fig. 4c; a partially formed semicircle at high frequencies, a semicircle at mid-frequencies, and a Warburg impedance tail at low frequencies. Qualitatively, the most notable change is the diameter of the mid-frequency semicircle (labelled mf Ø in Fig. 4c), which increases markedly for 4.2 and 4.3 V UCV. From three-electrode cell measurements on graphite/NMC811 cells after aging (Fig. S8†) it is evident that the NMC cathode is the main contributor to the observed full cell impedance rise. No change is observed in the full cell impedance after aging by CYC 2.5–3.8 V (see inset in Fig. 4c), consistent with the cell polarization result above. Area specific impedance (ASI) data from hybrid pulse power characterization (HPPC) tests was also collected before and after aging to measure the SOC dependent impedance rise. Plots of discharge ASI *versus* cell voltage for all cycling protocols are shown in Fig. S9.† For simplicity, the difference in ASI for the discharge pulse at ∼3.7 V before and after aging is plotted against UCV in Fig. 4d. This analysis confirms that aging by a VH at 3.8 V and CYC 2.5–3.8 V does not increase the cell impedance, and VH 4.2 V and VH 4.3 V see small changes after aging. HVC has resulted in a modest impedance rise of 8.8(6) Ω cm^2^. Full SOC changes combined with higher UCV appear to be the largest drivers for impedance rise, with CYC 2.5–4.2 V and CYC 2.5–4.3 V recording the largest increases of 11.1(8) and 29(2) Ω cm^2^, respectively. Note that while CYC 2.5–4.2 V has led to mild amounts of impedance rise (Fig. 4c and d) this evidently does not give rise to impedance-induced capacity loss in the DVA discussed above (Table S2†). On the contrary, the higher impedance rise measured for CYC 2.5–4.3 V does lead to capacity loss in the C/20 cycles analysed by DVA (Table 1).

### 3.5 NMC cathode capacity loss

**Fig. 5:**
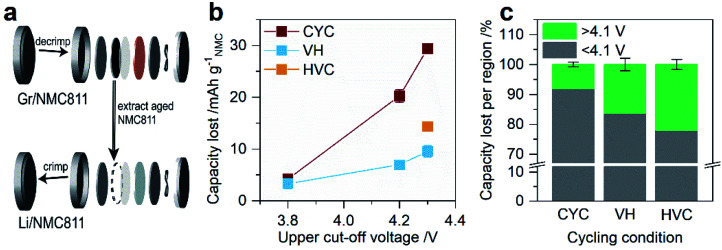
Aged NMC811 cathode discharge capacity retention and capacity loss after aging by constant current–constant voltage cycling (CYC), voltage hold (VH), and high voltage cycling (HVC). The capacity retention and loss are measured in Li/NMC811 half-cells with aged cathodes extracted from graphite/NMC811 full cells. Data is shown for the fifth C/20 cycle in the half-cell (3.0–4.5 V *vs.* Li/Li^+^) relative to the fifth C/20 cycle of a pristine (*i.e.* not aged in a full cell) NMC811 cathode cycled under the same conditions. (a) Schematic of aged cathode extraction and half-cell rebuild process. (b) Discharge capacity lost (in mA h g_NMC_^−1^) as a function of upper cut-off voltage. (c) Percentage charge capacity lost above and below 4.1 V *vs.* Li/Li^+^ for cells with a 4.3 V upper cut-off voltage. Error bars on the plots represent the spread of two or three duplicate cells.

After the aging and diagnostic cycles in the graphite/NMC811 full cells, the cells were decrimped and the cathode was harvested. Li/NMC811 half-cells with the aged cathodes were immediately built and cycled five times in the potential window 2.5–4.5 V *vs.* Li/Li^+^ – a schematic of the decrimp–rebuild process is shown in Fig. 5a. In the half-cell, the NMC is the capacity-limiting electrode on both charge (lithium removal) and discharge (lithium insertion) and therefore the difference in capacity between a pristine cathode (cycled in a half-cell under identical conditions) and the aged cathodes is a measure of the NMC capacity degradation. Representative potential profiles and differential voltage plots for the half-cells after equilibration (*i.e.* in the fifth C/20 cycle) are shown in Fig. S10,† which provide a comparison of the NMC capacity loss and over-potential after aging by the various aging protocols. Fig. 5b plots the capacity lost from NMC as a function of UCV and aging protocol. NMC has lost 3–4 mA h g_NMC_^−1^ of capacity after aging by CYC or VH with a 3.8 V UCV. Capacity lost increases with UCV, with the amount lost increasing in the following order: VH 4.2 V, VH 4.3 V, HVC, CYC 2.5–4.2 V, and CYC 2.5–4.3 V. Consistent with the DVA findings above, large SOC change combined with higher UCV appear to be the key drivers of NMC degradation. In Fig. 5c the percentage of capacity lost above and below 4.1 V *vs.* Li/Li^+^ is shown for protocols with a 4.3 V UCV, which isolates the effect of cycling condition on the NMC capacity loss at high voltage. The analysis uses the charge capacity, so the findings relate directly to the DVA in Fig. 3 above. This reveals that subjecting the NMC811 cathode to high voltage for a prolonged period, as in the VH 4.3 V and HVC protocols, leads to a higher fraction of capacity loss above 4.1 V *vs.* Li/Li^+^ as compared to cycling in a 2.5–4.3 V window. The larger fraction of high voltage capacity loss measured here for HVC is consistent with the result from DVA in Fig. 3e and f, which revealed a second high voltage-specific NMC capacity loss (termed NMC_HVdeg_) that was largely absent in the CYC 2.5–4.3 V condition.

### 3.6 Reduced surface layer

**Fig. 6:**
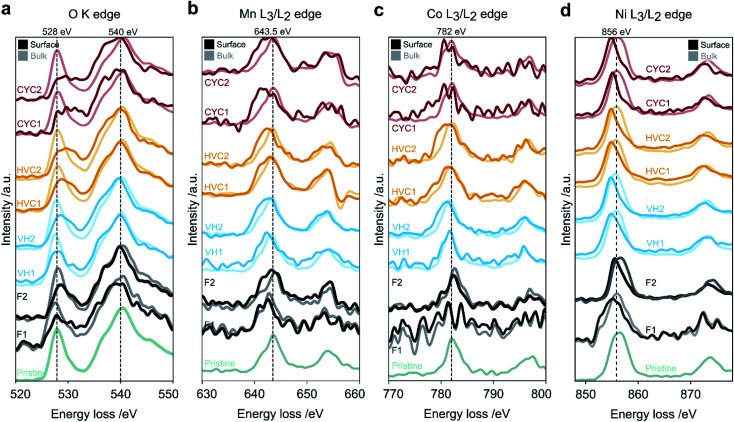
Average core-loss EEL spectra of pristine, formed (F), and aged NMC811 samples, with aging by constant current–constant voltage cycling (CYC 2.5–4.3 V), voltage hold (VH 4.3 V), and high voltage cycling (HVC). The spectra are arranged from bottom to top in increasing order of the amount of impedance rise (see Fig. 4d). Two spectra from different particles are shown for each condition, except the pristine sample. The fine structure EELS is shown for the (a) oxygen K edge, (b) manganese, (c) cobalt, and (d) nickel L_3_/L_2_ edges. Vertical dashed lines indicate relevant, expected peak positions and are shown as a guidance to the eye.^12^

To probe the oxidation states of the transition metals in aged NMC, we employed electron energy loss spectroscopy in a scanning transmission electron microscope (STEM-EELS), as it can probe local oxidation states and allow separation of the contribution of the surface and bulk regions thanks to its high spatial resolution. Core-loss EEL spectra of the surface and the bulk of particles aged by CYC, VH, and HVC protocols (all with an UCV of 4.3 V) and particles after the three C/20 formation cycles between 2.5–4.3 V (formed) are shown in Fig. 6. The spectra from the pristine and formed samples are shown as reference – the pristine sample does not have a pronounced layer of reduced transition metals at the surface (reduced surface layer, ReSL). This layer is developed during the first few cycles (shown by the formed sample) and the formation step is common to all protocols. Any further changes to the ReSL arising from aging can be compared to the state of the surface layer after formation. Before analysis, the aged cathodes were cycled in Li/NMC811 half-cells (see Section 3.5) to re-lithiate each electrode to an equivalent SOC corresponding to a fully lithiated (discharged) state. The different amounts of capacity loss from electrode slippage during aging (see Section 3.3) leave the aged NMC811 with different lithium content, even when the full cell is discharged, and therefore with different bulk TM oxidation states. After re-lithiation, any persisting changes to the bulk TM oxidation state can be identified by comparing the bulk spectra of aged and pristine particles.

For each cycling condition, two sets of spectra (surface and bulk) from different particles are shown. The spectra contain the K edge of oxygen and the L_3_/L_2_ edges of the three transition metals (TMs: Co, Mn, Ni). Details on the data processing steps taken and a brief description of the EELS methodology used can be found in ESI Note S3.†

**Fig. 7:**
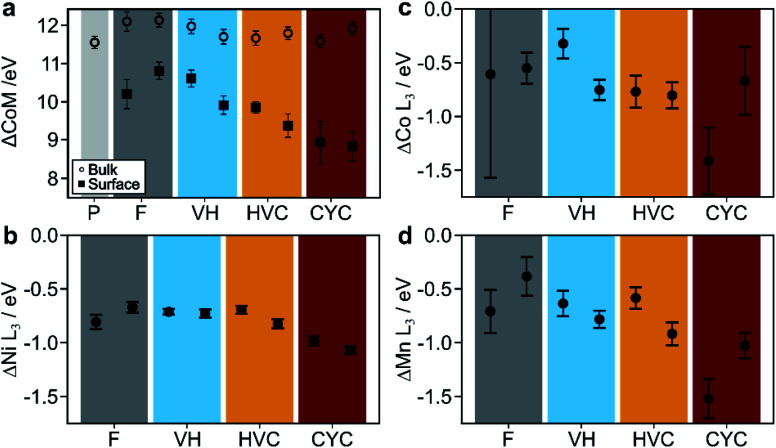
Results from EELS quantification. (a) Center of mass difference (ΔCoM) between the pre-edge peak and main peak of EELS O K-edge. (b–d) Ni, Co and Mn L_3_ peak position difference between the bulk and surface spectra (Fig. 6b–d). Colored blocks indicate samples aged by voltage hold (VH, blue), high voltage cycling (HVC, orange), and constant-current voltage cycling (CYC, red) protocols, each with UCV of 4.3 V. The sample after formation (F, dark grey) is shown for comparison. The pristine sample (P, light grey) is also shown for comparison in (a). It is not shown for (b–d) as there is no surface spectrum for the pristine sample. Duplicate data points come from two spectral images of different particles.

To compare the O K edge spectra for samples aged by different protocols, a model has been used to fit to every spectrum as described in ESI Note S3.† The difference (in eV) between the center of mass (ΔCoM) of the pre-edge peaks (at 528 and 531 eV) and the main edge peak (at 540 eV) was calculated for all surface and bulk spectra and shown in Fig. 7a. For the surface spectra, the ΔCoM parameter decreases in the following order: formed, VH, HVC, CYC. Such a trend is not seen in the bulk spectra, which remain largely unaffected by aging. This analysis indicates that the average TM oxidation state does not change significantly for the bulk material during the electrochemical protocols, while the degree of TM oxidation state reduction at the surface depends on the protocol.

As the O K-edge provides information on only the average oxidation state of all TMs, each of the L_3_/L_2_ edges of Co, Mn and Ni were analyzed in a similar manner (as described in ESI Note S3†). Briefly, the L_3_ peak of each edge was fitted with a single Gaussian function. The difference in peak positions between the surface and the bulk spectra (ΔTM L_3_) are shown in Fig. 7b–d. Starting with the Ni L edge (Fig. 6d and 7b), the peak position of the Ni L_3_ peak is at ∼856 eV for all the bulk spectra and shifts to ∼855 eV at the surface. Such behavior is consistent with the literature and is attributed to the reduction of nickel from Ni^3+^ to Ni^2+^ during the transformation of the initial layered structure to the rock salt phase (Fig. S11b†).^12^ Minor differences in ΔNi L_3_ are seen after aging by different protocols. The CYC condition has a slightly more reduced Ni oxidation state (*i.e.* larger ΔNi L_3_), while the other protocols result in similar chemical shifts. In contrast, the Co and Mn edges (Fig. 7c and d respectively) show more variation in ΔCo L_3_ and ΔMn L_3_ across the aging conditions. Specifically, the VH, HVC and CYC have larger ΔCo L_3_ and ΔMn L_3_, rising in that order, when compared to the formed sample. Larger chemical shifts signify higher degree of Co and Mn reduction at the surface, consistent with the trend of the O K-edge pre-edge peak broadening and shift (Fig. 7a). Exact quantification of the oxidation states of Co and Mn is difficult due to low signal-to-noise ratio. In summary, Ni appears to be reduced significantly, almost to Ni^2+^ during the formation cycles, with little change thereafter. Meanwhile, Co and Mn are further reduced at the surface, with the extent of reduction dependent on the aging protocol, indicating gradual surface degradation processes that are determined by the electrochemical history. Formation of the cell already introduces an ReSL, which then evolves further: the surface TMs are most reduced for the CYC, followed by HVC and VH protocols. This, combined with the impedance measurements introduced earlier, shows a correlation between the degree of average TM reduction in the ReSL and the amount of impedance rise caused by each protocol, as discussed in detail in the Discussion section.

### 3.7 Microstructure

**Fig. 8:**
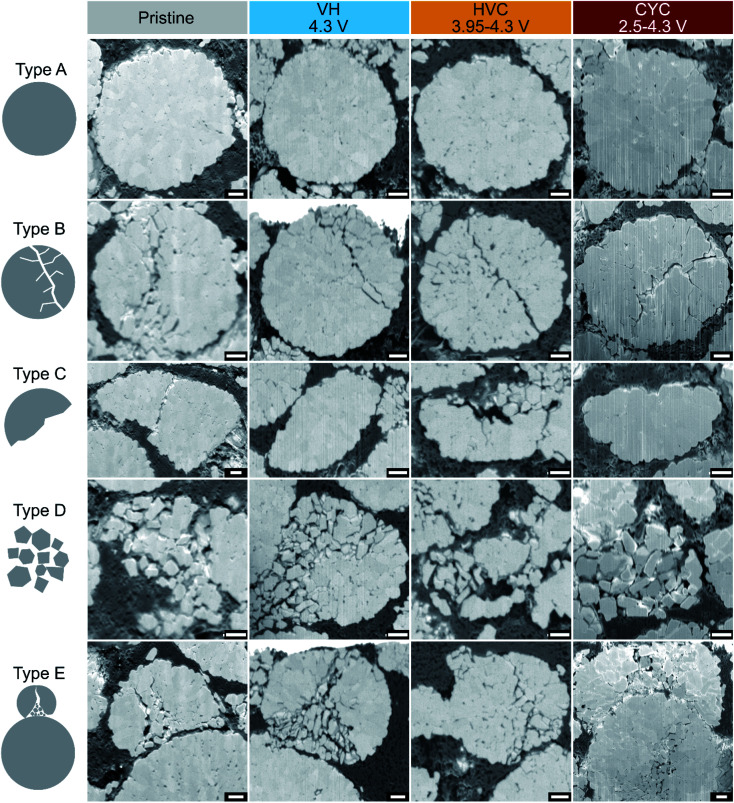
FIB-SEM tomography snapshots for a pristine NMC811 cathode and cathodes from cells aged by voltage hold (VH), high voltage cycling (HVC), and constant current–constant voltage cycling (CYC) with a 4.3 V upper cutoff voltage. Various types of microstructure can be identified: type A – non-defective secondary particles; and defective types formed as a result of the electrode fabrication process (type B–E). In all images the current collector side of the cathode is at the bottom and the scale bar is 1 μm.

The electrochemical protocols used in this work are designed to cause different unit cell volume changes (from 0 Å^3^ for VH to 5.5 Å^3^ for CYC with an UCV of 4.3 V, Fig. 1d–f) and the number of volume change cycles. As mentioned in the introduction, repetitive expansion and contraction of the lattice is thought to lead to mechanical cracking of NMC particles. Microstructure and the potential influence of the aging protocols on NMC particle cracking were investigated using dual-beam FIB-SEM tomography (focused ion beam-scanning electron microscope). A series of cross-sections were acquired from a pristine NMC811 cathode and cathodes aged by VH, HVC and CYC protocols with an UCV of 4.3 V. For each specimen, 200–400 slices were acquired, covering a volume of approx. 20 × 30 × 30 μm for each sample. A qualitative analysis highlights that regardless of the electrochemical history of samples (including the pristine, uncycled electrode), all electrodes appear to contain particles with various extents of cracking throughout the measured volume. Snapshots of the data are shown in Fig. 8, demonstrating the types of morphologies present. Akin to the work of Heenan *et al.*^25^ who categorize defective NMC particles in pristine electrodes, we observe the following types of microstructure in the pristine and cycled samples (pristine, VH, HVC, CYC with UCV of 4.3 V): secondary particles without any apparent microcracks (type A); secondary particles with significant cracking, mostly in a form of a single main crack and narrower side-cracks (type B); secondary particles that were fractured during synthesis or electrode fabrication, where only a part of the original spherical shape is seen (type C); material shattered into separate primary particle ‘rubble’ (type D); and lastly a microstructure that most clearly highlights the calendering as its cause in the form of two secondary particles in stacked close contact with visible damage due to local stress in the direction perpendicular to the plane of the current collector (vertical in the image, type E). The results from FIB-SEM tomography show that intergranular cracking (between primary particles) is extensive in all cathodes used in this work due to the calendering process, which is the industry standard.^25^

## 4. Discussion

The results in this work indicate that there are three main processes that lead to capacity loss in graphite/NMC811 full cells: capacity loss from electrode slippage, impedance, and NMC cathode degradation. In the context of this work, electrode slippage is quantified using DVA by analyzing the third C/20 formation cycle and the third C/20 diagnostic cycle after aging. As such, capacity loss arising from the formation of the SEI (which mostly takes place in the first cycle(s) of the cell) is not captured in this analysis allowing us to focus on the losses due to ongoing SEI repair made necessary by the electrochemical aging protocol applied. Capacity loss from electrode slippage is detected after all aging protocols (*i.e.* CYC, VH, and HVC) irrespective of UCV, change in SOC, and unit cell volume change. It is the largest contributor to capacity loss after aging by CYC (≥70% of the measured capacity loss – see Tables 1 and S2†), in agreement with prior literature for NMCs with 33, 50, and 80% Ni content.^21–24,26–28^ Electrode slippage is found to be accelerated by increasing the cell UCV during cycling. This is consistent with prior reports in which lattice oxygen loss, and the connected electrolyte solvent oxidation, and TM dissolution from NMC are found to increase rapidly at high SOCs,^23,29,30^ coupled with other reports that have demonstrated that TMs and solvent degradation species disrupt the graphite SEI and lead to enhanced electrolyte decomposition (which diminishes cyclable lithium) and impedance rise.^23,26,31–40^ Interestingly, while the total capacity delivered over the aging period for CYC 2.5–4.3 V and HVC are similar (28.8(1) A h g_NMC_^−1^ and 29.7(7) A h g_NMC_^−1^, respectively), the capacity loss and electrode slippage are lower for the HVC protocol (see Fig. 2 and Table 1), highlighting the important role of the electrochemical protocol in driving electrode slippage. For aging by VH, electrode slippage is the sole mechanism leading to capacity fade, indicating that while SOC changes accelerate electrode slippage, cycling is not a requirement for lithium loss by this process.

The second degradation process identified is cell impedance rise. As shown in Fig. 4d, aging by CYC with UCV ≥4.2 V and HVC (3.95–4.3 V) give rise to the highest amounts of impedance rise (9–29 Ω cm^2^). VH protocols and CYC with a 3.8 V UCV gave rise to negligible or small (0–4 Ω cm^2^) impedance increase, reinforcing the conclusion that electrode SOC changes coupled with high UCV are the main drivers for impedance rise. The low impedance measured after VH at 4.2 and 4.3 V also indicates that electrolyte degradation reactions leading to CEI formation at the NMC surface, which are accelerated at higher voltage,^16,41^ are not the primary source of cell impedance, consistent with prior reports.^42^ It is interesting to note that aging by CYC to 4.2 V and HVC (3.95–4.3 V) give rise to similar impedance increases – 11.1(8) and 8.8(6) Ω cm^2^, respectively, in Fig. 4d. Despite having rather different NMC ΔSOC (71% for CYC 2.5–4.2 V and 17% for HVC 3.95–4.3 V at the start of the aging protocol), the NMC unit cell volume change within the respective voltage ranges are similar at 4.5 and 4.1 Å^3^, respectively. This may suggest a link between the abrupt NMC lattice contraction and impedance rise. To rationalize this link, a number of interconnected processes must be considered. First, the structural changes induced by cycling have been demonstrated to promote inter-particle cracking^4,6,7,43–45^ (evident in the charged state even in the first cycle),^46–48^ which leads to increased electrolyte accessibility within the secondary particle and enabling the formation of an O-depleted resistive surface layer (and reduced surface layer) on the primary particle surface, as proposed recently by Friedrich *et al.*^54^ and Zou *et al.*^15^ This layer hinders the transport of lithium ions and/or electrons into the particle resulting in impedance rise.^12,49^ Our analysis of the particle cracking and the reduced surface layer is discussed below.

At this point, it is important to make the distinction between reversible and irreversible capacity losses. Capacity loss from electrode slippage is irreversible since the lithium ions are immobilized in the graphite SEI and no longer shuttle from cathode to anode. Conversely, the amount of capacity “lost” from impedance is dependent on the applied current and can be recovered with a sufficiently slow cycling rate, *i.e.* when the associated overpotential becomes negligible – see ESI Note S4.† Irrespective of the aging protocol, at a C/20 cycling rate very small capacity losses (<2 mA h g_NMC_^−1^) were attributed to impedance rise, in agreement with the results in Fig. S12† and reported previously.^54^

The final capacity loss process is degradation of the NMC811 cathode material. To isolate the capacity fading mechanisms of Ni-rich NMC cathodes from the loss of cyclable lithium taking place at the graphite interphase (manifesting as capacity loss by electrode slippage), many researchers resort to cycling half-cells with a lithium metal anode.^3,4,6,44,50,51^ However, electrolyte degradation products formed at the reactive lithium metal surface can cross the separator and react at the NMC surface (termed cross-talk) leading to degradation that is not representative of the full cell chemistry.^52,53^ Others have built and cycled full cells with an electrochemically pre-lithiated graphite anode,^54^ in which the ongoing lithium losses due to SEI repair are compensated by the large excess of lithium initially on the graphite electrode. In this work we use DVA to differentiate the capacity fading of Ni-rich NMC from the loss of cyclable lithium due to SEI formation and repair, which allows us to quantitatively decouple these capacity loss mechanisms in a standard full cell format. The capacity loss term NMC_deg_ defined above quantifies the capacity lost evenly across the active SOC of the NMC cathode. (Note that electrode slippage decreases the capacity utilization of the NMC without necessarily resulting in NMC degradation.) Aging by VH gave rise to negligible (<1 mA h g_NMC_^−1^) capacity loss attributed to NMC_deg_, suggesting that time spent at high voltage is not a key contributor. Conversely, NMC_deg_ is an active mode of capacity loss in CYC 2.5–4.2 V, CYC 2.5–4.3 V, and HVC protocols, accounting for 16–27% of the measured capacity loss. Half-cell measurements with aged NMC811 cathodes extracted from full cells (Fig. 5) support these findings. As such, we find that NMC degradation is driven by NMC SOC changes coupled with high UCV, which are the same electrochemical stimuli that promote impedance rise. This may indicate that NMC degradation and impedance rise are linked. Certainly, the explanation given above for impedance caused by formation of a resistive reduced surface layer on the NMC particles, which is electrochemically inactive, is consistent with this hypothesis.

Using DVA we also detect a high voltage NMC degradation mechanism leading to capacity loss at potentials >4.1 V *vs.* Li/Li^+^ (Fig. 3e and f). The post-test half-cell experiments with aged NMC811 cathodes (Fig. 5c) also provide support for this aging process. While further investigation is required to fully understand the mechanism(s) leading to high voltage NMC degradation, it could be related to the observations recently made by Xu *et al.*^55^ In their work, long-duration operando synchrotron X-ray diffraction of graphite/NMC811 full cells revealed that after repeated cycling a fraction of the NMC material could not be delithiated beyond a SOC of approximately 75%, leading to “active” and “fatigued” phases. This was ascribed to a “structural pinning” at potentials ≥4.2 V *vs.* Li/Li^+^. Our work clearly indicates that high voltage NMC degradation is driven by subjecting the NMC811 cathode to high voltage for a prolonged period (*i.e.* 750 h in the 4.3 V voltage hold protocol) but even more so by a large number of cycles through the high voltage range, as in the HVC protocol (∼800 cycles between 3.95–4.3 V). Therefore, we propose that capacity loss *via* this mechanism is not evident in the DVA for CYC 2.5–4.3 V due to the smaller number of cycles (*i.e.* 150) and less time spent at high voltage.

Particle cracking is often identified as an important degradation process for NMC cathodes,^43–45^ which we investigate in this work using FIB-SEM tomography. It seems, however, that the material synthesis and electrode fabrication process – in particular electrode calendering (as demonstrated by FIB-SEM tomography results in Fig. 8) – are much more destructive at the microscale to the NMC particles than the electrochemical stimuli during aging. Indeed, even in the pristine, uncycled electrode it is easy to find NMC secondary particles that are cracked or even shattered into primary particles (Fig. 8), consistent with the recent work of Heenan *et al.*^25^ in which X-ray nano-computed tomography (CT) is used to quantify the number of defective particles within a commercial NMC811 electrode. These authors determine that approximately one-third of particles in the pristine electrode are cracked and/or shattered. The vast extent of particle damage in the pristine electrode makes it challenging to distinguish any electrochemically induced microscale particle cracking in the aged electrodes in the discharged state. This is despite the harsh conditions that the NMC cathode was subjected to in the HVC protocol, *i.e.* ∼800 cycles between 3.95–4.3 V with a large 4.1 Å^3^ NMC unit cell volume change per cycle. Recently, several groups have shown evidence of microscale cracking of NMC particles imaged in the charged state in the first cycle.^46–48^ After the first discharge, however, the cracks are no longer observed^46^ suggesting a reversible (at least initially) “breathing” of the NMC particles. In this work, electrochemically induced cracks are not visible in NMC particles imaged in the discharged state (Fig. 8) suggesting that the particles continue to reversibly expand and contract after these aging protocols. We propose that the high performance of calendered commercial electrodes (in terms of high capacity and low impedance) indicates that mechanical cracking and/or pulverization of a significant portion of NMC secondary particles does not immediately give rise to capacity loss or impedance rise – see further discussion in ESI Note S5.† However, it is important to note that defects induced by either electrode manufacture or the electrochemical protocol may initiate degradation processes that manifest themselves in terms of capacity loss and impedance rise over the course of longer-term electrochemical aging. It is also possible, that electrochemical stimuli influence the structure of the electrodes at smaller length scales than FIB-SEM tomography can measure (*i.e.* intragranular cracking, within primary particles) as suggested by some reports,^50,51^ and the impact of these on the capacity retention is not currently known. An investigation to examine such mechanisms is beyond the scope of the current work.

As the results discussed above point towards the reduced surface layer as a major contributor to impedance rise in graphite/NMC811 cells, we investigated the chemistry of that layer, by probing the oxidation states of TMs at the surface and in the bulk of the NMC samples aged by different protocols using STEM-EELS. Our comprehensive analysis of EELS spectrum images (as opposed to line or point scans, which are typical in the literature) allowed us to explore the oxidation states of all three TMs in relation to the cycling protocol applied. In our analysis, we omitted analysis of the thickness of the ReSL on purpose for a number of reasons. First, it is challenging to measure reliably because of projection artefacts (*e.g.* thinner samples lead to apparent thicker ReSL, see Fig. S13†). Second, a report by Li *et al.*^56^ shows that the thickness of the ReSL is heavily dependent on the electrolyte additives and is not well correlated with the impedance rise. Finally, the thickness of the reconstructed surface layer is suggested to be facet dependent, with larger thickness on the facets that are permeable to lithium, as demonstrated for single crystalline NMC333 and NMC622 in a recent report by Zhu *et al.*^57^ Therefore, we focus on the chemical nature of the ReSL rather than its thickness. Our results indicate that the ReSL appears already during the first few cycles (formation), which is consistent with the literature.^58–60^ However, our work also shows that the ReSL does not reach its final state immediately. Instead, initial cycles lead to reduction of Ni, while Co and Mn only show negligible changes to their oxidation states. During further cycling, the TM oxidation states of the surface layer are reduced further, with the biggest changes to Co and Mn. The degree of average TM reduction is the lowest for the formed sample, and increases for VH, HVC and CYC samples (in that order). TM reduction must be accompanied by oxygen release from the surface of particles in order to maintain charge neutrality, leading to a structural transformation from layered to spinel or rock salt-like phases. Jung *et al.*^9^ show that the amount of oxygen release is largest during the first cycle and decreases rapidly in the following cycles. Our EELS results agree with that observation, as the largest changes in TM oxidation states at the surface are found between the pristine and formed samples, with more gradual evolution after formation. As demonstrated in Fig. 4d, 6, 7 and S9,† the amount of impedance rise is correlated with the degree of reduction of TMs at the surface of particles, with higher impedance corresponding to more severe Co and Mn reduction. The oxidation state of Ni reaches its saturation (Ni^2+^) more readily and only shows small differences between the aging protocols. The progressive reduction of Ni, and then Co and Mn, is consistent with the relative stabilities (and redox couples) of the M^4+^ and M^2+^ ions. Note that the formation of Mn^2+^ and Co^2+^ ions may also result in increased dissolution of the NMCs. Therefore, we propose that the full cell impedance rise is mostly due to changes to the structure and chemistry of surface layer on the NMC particles, where Ni reduction happens and is detectable first and, as the degradation progresses further, the TMs (especially Co and Mn) on the surface are gradually reduced further (accompanied by oxygen release). As an all M^2+^ rock salt (MO) structure is unlikely to contain Li^+^ ions or cation vacancies, the increasing metal reduction observed (changing the structure towards the rock salt) is expected to cause increased impedance due to poorer lithium transport through the surface layer. To the best of our knowledge, such behavior has not been previously reported.

## 5. Conclusions

By using strategically designed electrochemical protocols, we have determined that the main causes for capacity loss in graphite/NMC811 lithium-ion batteries are lithium inventory loss due to electrode slippage, impedance rise, and NMC cathode degradation. By varying the protocol conditions, such as upper cutoff voltage, time at high voltage, change in state of charge and NMC unit cell volume, and the number of cycles, we were able to link the capacity loss to particular electrochemical stimuli. Larger state of charge and NMC unit cell volume changes coupled with high upper cutoff voltage are the main drivers for full cell impedance rise and NMC capacity loss. We propose that both of these are primarily caused by the formation of the electrochemically inactive and insulating reduced surface layer on the NMC particles. Comprehensive STEM-EELS analysis reveals that the chemistry of the reduced surface layer evolves throughout aging and the degree of TM reduction, particularly Mn and Co, is correlated with higher impedance rise and a more resistive surface layer. Our analysis using FIB-SEM tomography indicates that electrode manufacture, and in particular calendering, is far more destructive than electrochemical induced microscale particle cracking and introduces extensive particle damage even before cycling begins. Overall, our results indicate that the extent and types of degradation are directly linked to the electrochemical cycling protocol. Advanced understanding of these relationships is critical to enable the development of high energy Ni-rich lithium-ion batteries.

## Author contributions

Wesley M. Dose and Jędrzej K. Morzy: conceptualization, methodology, formal analysis, investigation, writing – original draft. Amoghavarsha Mahadevegowda: investigation, writing – review & editing. Caterina Ducati, Clare P. Grey, and Michael F. L. De Volder: conceptualization, writing – review & editing, supervision, funding acquisition.

## Conflicts of interest

There are no conflicts to declare.

## Supplementary Material

TA-009-D1TA06324C-s001
