# Supplementary material for: The influence of electrochemical cycling protocols on capacity loss in nickel-rich lithium-ion batteries
Source: J Mater Chem A Mater. 2021 Oct 11;9(41):23582–96. doi: 10.1039/d1ta06324c (PMC8547555; doi:10.1039/d1ta06324c)
Supplement: TA-009-D1TA06324C-s001 [file TA-009-D1TA06324C-s001.pdf]

Electronic Supplementary Material (ESI) for Journal of Materials Chemistry A.
This journal is © The Royal Society of Chemistry 2021

Supplementary information

The influence of electrochemical cycling protocols on capacity loss in nickel-rich lithium-ion batteries

Wesley M. Dose[1,2,4], Jędrzej K. Morzy[1,3,4], Amoghavarsha Mahadevegowda[3,4], Caterina Ducati[3,4], Clare P. Grey[2,4]*, Michael F. L. De Volder[1,4]*

[1]Department of Engineering, University of Cambridge, 17 Charles Babbage Road, CB3 0FS, Cambridge, UK
[2]Department of Chemistry, University of Cambridge, Lensfield Road, Cambridge, CB2 1EW, Cambridge, UK
[3]Department of Materials Science and Metallurgy, University of Cambridge, 27 Charles Babbage Road, CB3 0FS, Cambridge, UK
[4]The Faraday Institution, Quad One, Harwell Science and Innovation Campus, Didcot OX11 0RA, UK

*Correspondance: Michael F. L. De Volder (mfld2@cam.ac.uk), Clare P. Grey (cpg27@cam.ac.uk)

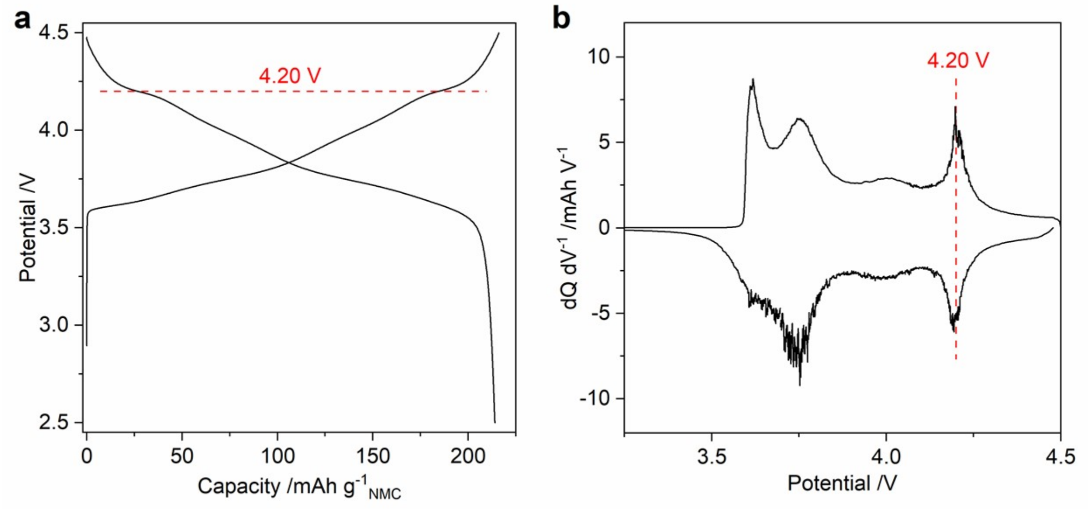

Figure S1. Representative (a) potential profile and (b) differential voltage plot for the fifth C/20 cycle (3.0-4.5 V vs. Li/Li^+) of a Li/NMC811 half-cell. The process at 4.2 V vs Li/Li^+ on charge and discharge is indicated.

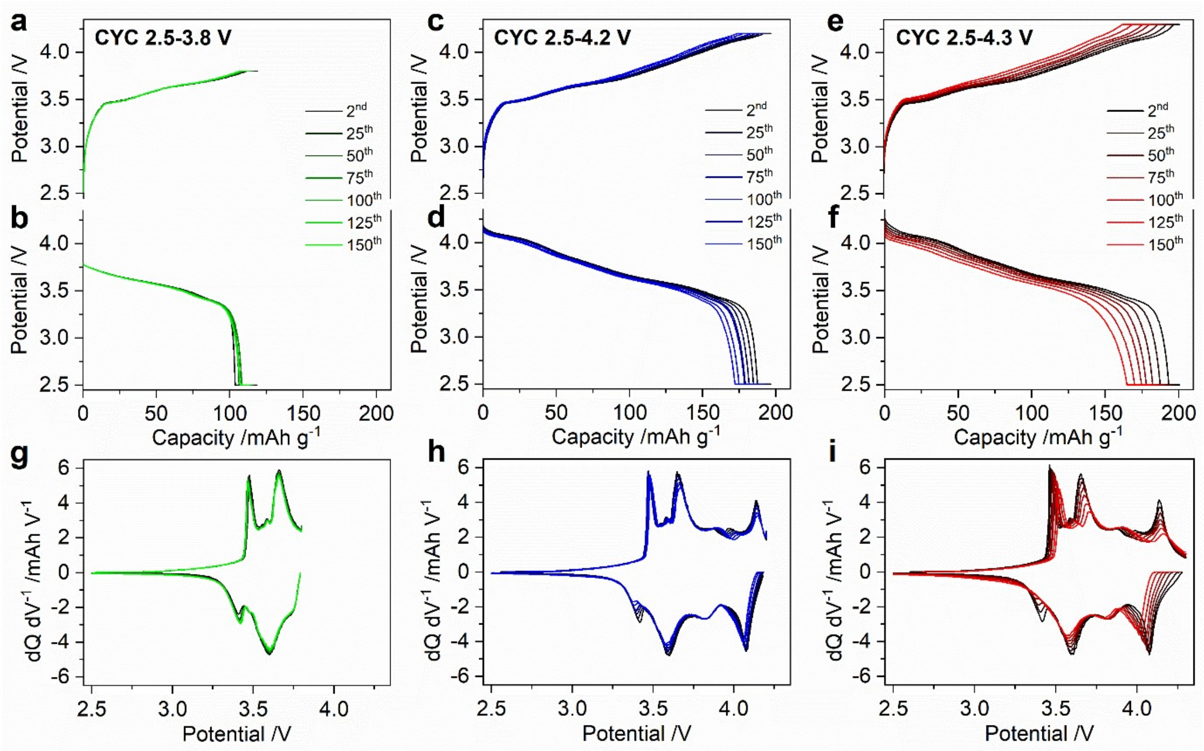

Figure S2. (a-f) Representative full cell voltage profiles and (g-i) differential voltage plots during aging by constant current-constant voltage cycling (CYC) at C/2 for UCV 3.8 V (a, b, g), 4.2 V (c, d, h), and 4.3 V (e, f, i). Every 25th cycle is shown.

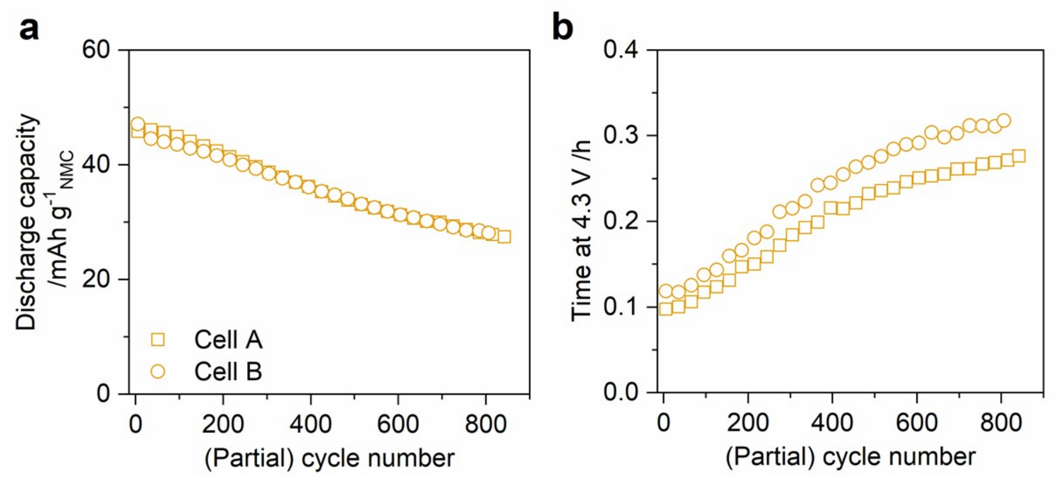

Figure S3. Full cell graphite/NMC811 performance during HVC aging cycles: (a) discharge capacity and (b) time on the 4.3 V constant voltage charge step. Data for two duplicate cells are shown separately. Due to the narrow voltage window (3.95-4.3 V), the cells completed >800 partial cycles during the 750 h aging period – the partial cycle count for the two duplicate cells is 801 and 837.

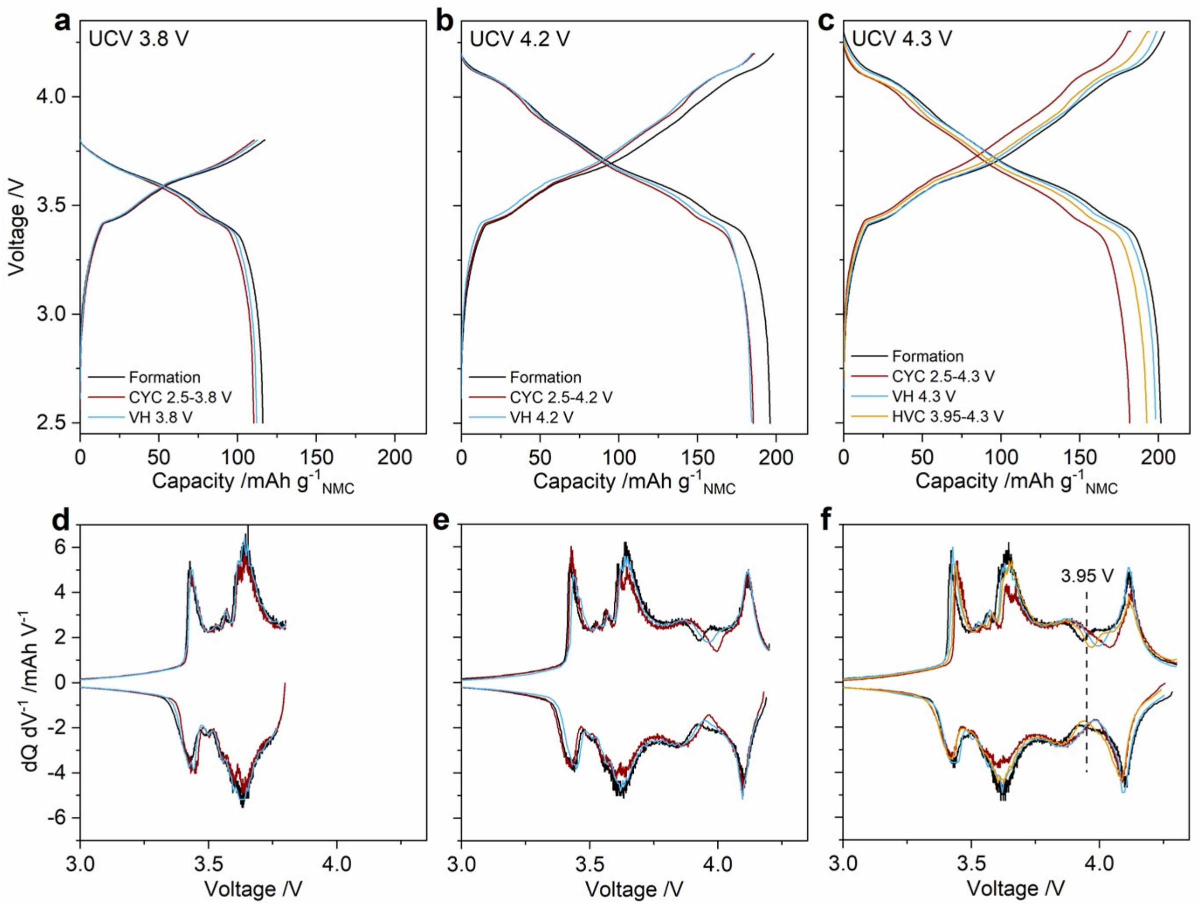

Figure S4. Representative full cell (a-c) voltage profiles and (d-f) differential voltage plots of the third C/20 formation cycle compared with the third C/20 diagnostic cycle. Aging by constant current-constant voltage cycling (CYC), voltage hold (VH), and high voltage cycling (HVC). Data is grouped by upper cutoff voltage, with 3.8 V in (a, d), 4.2 V in (b, e), and 4.3 V in (c, f).

Supplementary Note S1: Differential voltage analysis

Side reactions at the anode and/or cathode cause the capacity alignment of the electrodes in a full cell to shift relative to their alignment at the start of testing, a phenomenon termed electrode slippage. For example, electrolyte reduction at the graphite electrode immobilizes lithium ions in the solid electrolyte interphase (SEI), which diminishes the active lithium inventory of the cell leading to capacity loss. Differential voltage analysis (DVA), first reported by Bloom et al.[1–3], is a method to track and quantify the slippage in a full cell without the need of a third reference electrode, which often leads to poorer and less representative full cell electrochemistry. The voltage of a full cell, V_{cell}, can be expressed in terms of the voltage of the cathode, $V_{cathode}$, and anode, V_{anode}, by

$$V_{cell} = V_{cathode} - V_{anode} \qquad \text{(S1)}$$

Taking the derivative of the voltage with respect to capacity yields

$$\left(\frac{dV}{dQ}\right)_{cell} = \left(\frac{dV}{dQ}\right)_{cathode} - \left(\frac{dV}{dQ}\right)_{anode} \qquad \text{(S2)}$$

The expression in Equation S2 shows that dV/dQ contributions from the cathode and anode electrodes sum linearly to give the dV/dQ of the cell. Therefore, DVA involves fitting half-cell anode and cathode dV/dQ data to the full cell dV/dQ data and allowing the relative capacity alignment to change. Additional parameters can also be introduced, as necessary, to account for other processes that affect the voltage-capacity profiles. For example, capacity loss terms for the cathode and anode can be included to quantify the loss of active sites for ion insertion at each electrode. These terms apply a scalar multiplier to the half-cell capacity, effectively compressing the dV/dQ curve horizontally. Therefore, the results from the DVA fitting quantify the capacity loss attributable to degradation at the cathode, anode, and from electrode slippage, decoupling the contribution of each from the measured full cell capacity loss.

To illustrate the approach taken in this work, a full cell voltage profile during charge at C/20 is plotted versus capacity in Figure S3, together with the capacity normalized differential

voltage (Q dV/dQ). The full cell data shown in Figure S5 is for a cell aged by constant current-constant voltage cycling (CYC) in a 2.5-4.2 V window. Half-cell data collected separately in Li/NMC811 and Li/graphite half-cells are also shown. A linear combination of the dV/dQ half-cell data as per Equation S2 generates the full cell model profile, which is fitted to the full cell raw data using a least squares approach. Three parameters are allowed to refine during the data fitting: electrode slippage, cathode capacity loss, and anode capacity loss. By comparing the fitted parameters for the diagnostic cycle after aging with those in the formation cycle before aging, the amount of capacity loss arising from each of the above listed processes can be quantified. For the cell shown in Figure S5, the fitted parameters for before and after aging are listed in Table S1, together with their conversion into capacity loss terms with units in mAh $g^{-1}{}_{NMC}$. Finally, based on the fitting result, the features in the full cell dV/dQ profile in Figure S5b are assigned as originating from either the cathode (c_n) or anode (a_n), which highlights which electrode(s) dominate(s) the voltage profile in a given region.

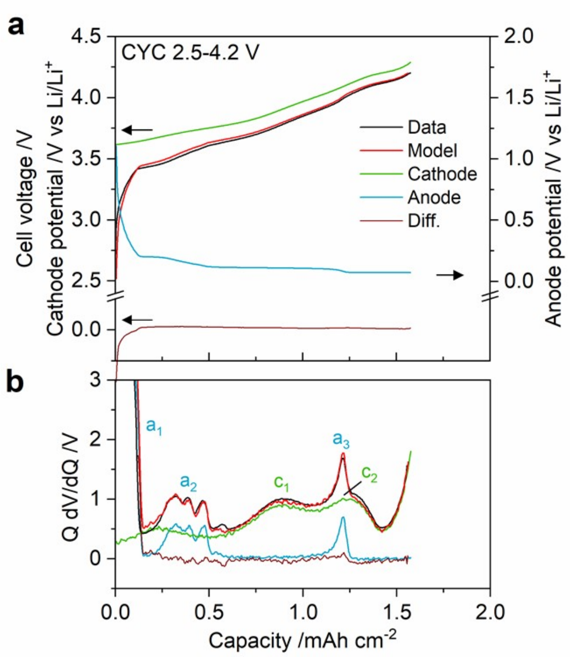

Figure S5. Example of fit from differential voltage analysis. (a) Charge voltage profile and (b) differential voltage versus capacity for the third C/20 diagnostic cycle after aging by CYC 2.5-

4.2 V (black), together with the modelled profiles from differential voltage analysis (red), the difference between the model and data (brown), and the NMC811 cathode (green) and graphite anode (blue) half-cell potential profiles used to generate the full cell model profiles. The derivative, (dV/dQ), in (b) is multiplied by the cell capacity (Q) in the given cycle, which normalizes the derivative based on the cell capacity. In (b), features in the dV/dQ profile are assigned to cathode (c_n) or anode (a_n).

Table S1. Fitted parameters from differential voltage analysis. Slippage and electrode degradation fitted parameters (in mAh g^{-1}_{NMC}) are listed for the formation and diagnostic cycles before and after aging by CYC 2.5-4.2 V.

Cycle	Slippage (mAh g^{-1}_{NMC})	NMC_{deg}* (mAh g^{-1}_{NMC})	Gr_{deg}* (mAh g^{-1}_{NMC})
Formation	0 (arb.)	0 (arb.)	0 (arb.)
Diagnostic / Capacity lost	9.5(4)	3.7(3)	2.3(1)^

* NMC_{deg} and Gr_{deg} are the fitted capacity lost attributable to degradation of the NMC cathode and graphite anode, respectively.
^ In this case, Gr_{deg} is determined to be 2.3 mAh g^{-1}, however, this apparent decrease does not affect the full cell capacity since the storage capacity of the graphite anode is not limiting. It is also worth noting that in all other conditions (e.g. VH 4.2 V, VH 4.3 V, CYC 2.5-4.3 V, HVC) Gr_{deg} is found to be <0.8 mAh g^{-1} suggesting little, or no degradation of the graphite under the cycling conditions employed in this work.

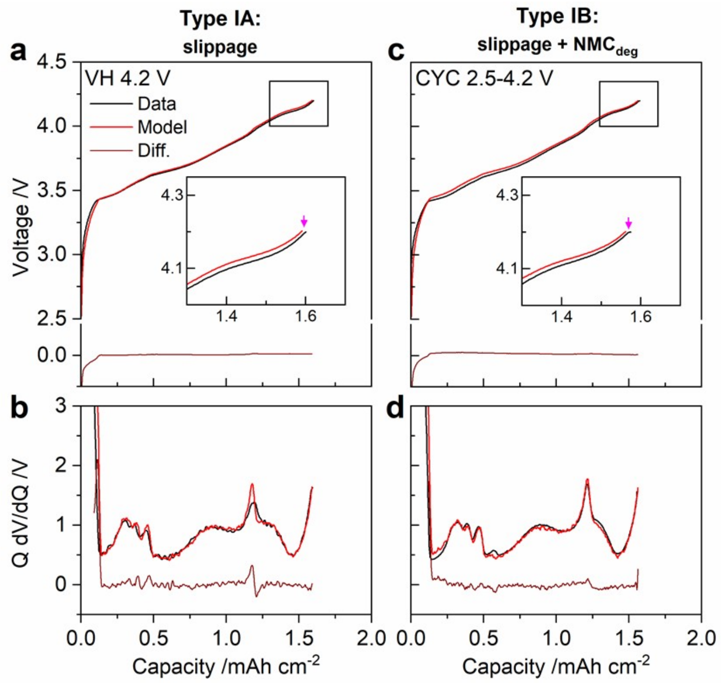

Figure S6. Identification of capacity loss mechanisms using differential voltage analysis for VH 4.2 V and CYC 2.5-4.2 V. (a, c) Charge voltage profiles and (b, d) differential voltage versus capacity for the third C/20 diagnostic cycle (black) together with the modelled profiles from differential voltage analysis (red), and the difference between model and data (brown). The insets in (a, c) show a magnified view of the profiles nearing the end of charge. The derivative, (dV/dQ), in (b, d) is multiplied by the cell capacity (Q) in the given cycle, which normalizes the derivative based on the cell capacity. Magenta arrows highlight the closely modelled charge capacity (down arrow).

Table S2. Breakdown of capacity loss mechanisms determined by differential voltage analysis (DVA) for all conditions analysed. Modelled and measured capacity lost for the examples highlighted in Figure 3 and in Figure S6 are given. The capacity lost is measured between the third C/20 formation cycle and after aging in the third C/20 diagnostic cycle.

	**Type IA:** slippage		**Type IA:** slippage		**Type IB:** slippage + NMC_{deg}		**Type II:** slippage + impedance + NMC_{deg}		**Type III:** slippage + impedance + NMC_{deg} + NMC_{HVdeg}	
Aging condition	VH 4.2 V		VH 4.3 V		CYC 2.5-4.2 V		CYC 2.5-4.3 V		HVC	
Degradation mode	Capacity lost (mAh g^{-1}_{NMC})	Est. % overall	Capacity lost (mAh g^{-1}_{NMC})	Est. % overall	Capacity lost (mAh g^{-1}_{NMC})	Est. % overall	Capacity lost (mAh g^{-1}_{NMC})	Est. % overall	Capacity lost (mAh g^{-1}_{NMC})	Est. % overall
Slippage†	7.5(6)	87	4(1)	90	9.5(4)	69	17.6(3)	77	2(2)	18
Impedance (C/20)^	0.8	9	0.3	5	0.5	4	1.7	7	1.2	11
NMC_{deg}†	0.4(5)	4	0(2)	5	3.7(3)	27	4(1)	16	3(3)	23
NMC_{HVdeg}‡			-	-			-	-	5(1)	48
Total modelled	9(1)	100	5(3)	100	13.7(7)	100	24(1)	100	11(6)	100
Measured*	8.8		4.6		13.3		23.2		11.0	

† Capacity from electrode slippage and NMC_{deg} were determined from DVA. NMC_{deg} is the fitted capacity lost attributable to degradation of the NMC cathode.

^ Capacity lost due to impedance is taken as the difference in the CV charge capacity for the formation and diagnostic cycles, which both have a C/40 cutoff current.

‡ NMC_{HVdeg} is the NMC cathode capacity loss that occurs specifically at NMC potentials >4.1 V vs Li/Li^+, and is in addition to NMC_{deg} which is lost evenly across the entire SOC. More details on how NMC_{HVdeg} is calculated are provided in Supplementary Note S2.

* Measured capacity loss is the difference in the charge capacity at the end of the CC segment for the formation and diagnostic cycles, at a C/20 rate.

Supplementary Note S2: Determining capacity lost due to NMC high voltage degradation

Aging by high voltage cycling (HVC 3.95-4.3 V) leads to a capacity mismatch between the data and the model derived from DVA (see Figure 3e, f and S5), with the model over-estimating the true capacity after aging. The model deviates from the data only at high SOC, where the NMC cathode dominates the voltage and dV/dQ profiles, suggesting a second NMC capacity loss process occurs specifically at NMC potentials >4.1 V vs Li/Li^+ – see Figure S5. This is capacity loss process is termed NMC high voltage degradation (NMC_{HVdeg}). Note that this is in contrast to NMC_{deg}, which models capacity lost evenly across the entire SOC range. Thus capacity lost from NMC_{deg} and NMC_{HVdeg} are additive. The amount of capacity lost due to NMC_{HVdeg} is determined by taking the difference in the modelled capacity and the measured CCCV capacity. The modelled capacity accounts for capacity lost from slippage and NMC_{deg} and that on the CV step accounts for capacity lost due to impedance. We attribute the remainder to NMC_{HVdeg}.

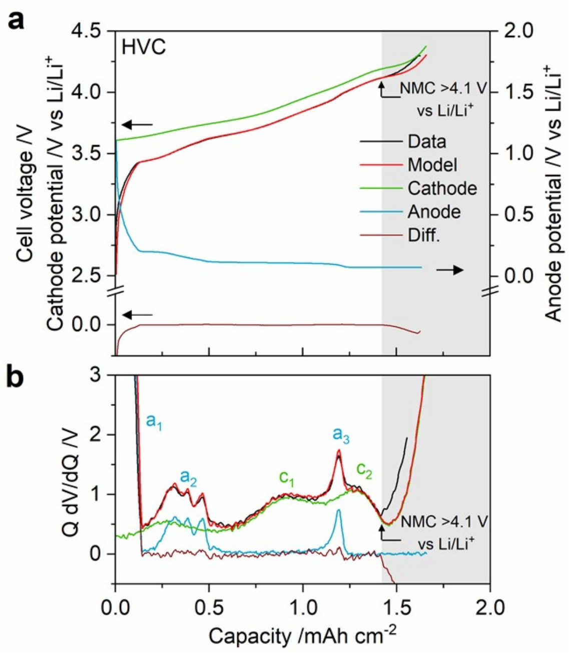

Figure S7. Fit from differential voltage analysis after aging by HVC. (a) Charge voltage profile and (b) differential voltage versus capacity for the third C/20 diagnostic cycle after aging by HVC (black), together with the modelled profiles from differential voltage analysis (red), the

difference between the model and data (brown), and the NMC811 cathode (green) and graphite anode (blue) half-cell potential profiles used to generate the full cell model profiles. The derivative, (dV/dQ), in (b) is multiplied by the cell capacity (Q) in the given cycle, which normalizes the derivative based on the cell capacity. In (b), features in the dV/dQ profile are assigned to cathode (c_n) or anode (a_n). The grey shaded region highlights where the model deviates from the data at high SOC, which begins when the NMC cathode is at 4.1 V vs Li/Li^+.

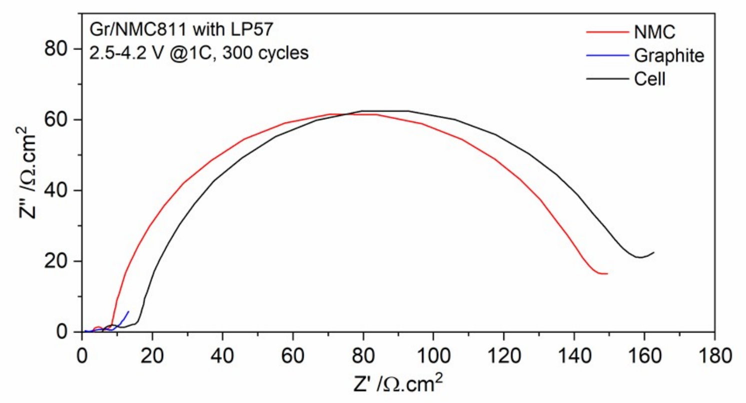

Figure S8. Nyquist plot with electrochemical impedance spectra of the full cell, NMC811 cathode, and graphite anode. Spectra of the NMC811 cathode and graphite anode were measured versus the Li metal reference electrode in a three-electrode EL-cell at 25 °C. The spectra were measured at a full cell voltage of 3.8 V after 300 aging cycles at 1 C rate between 2.5-4.2 V.

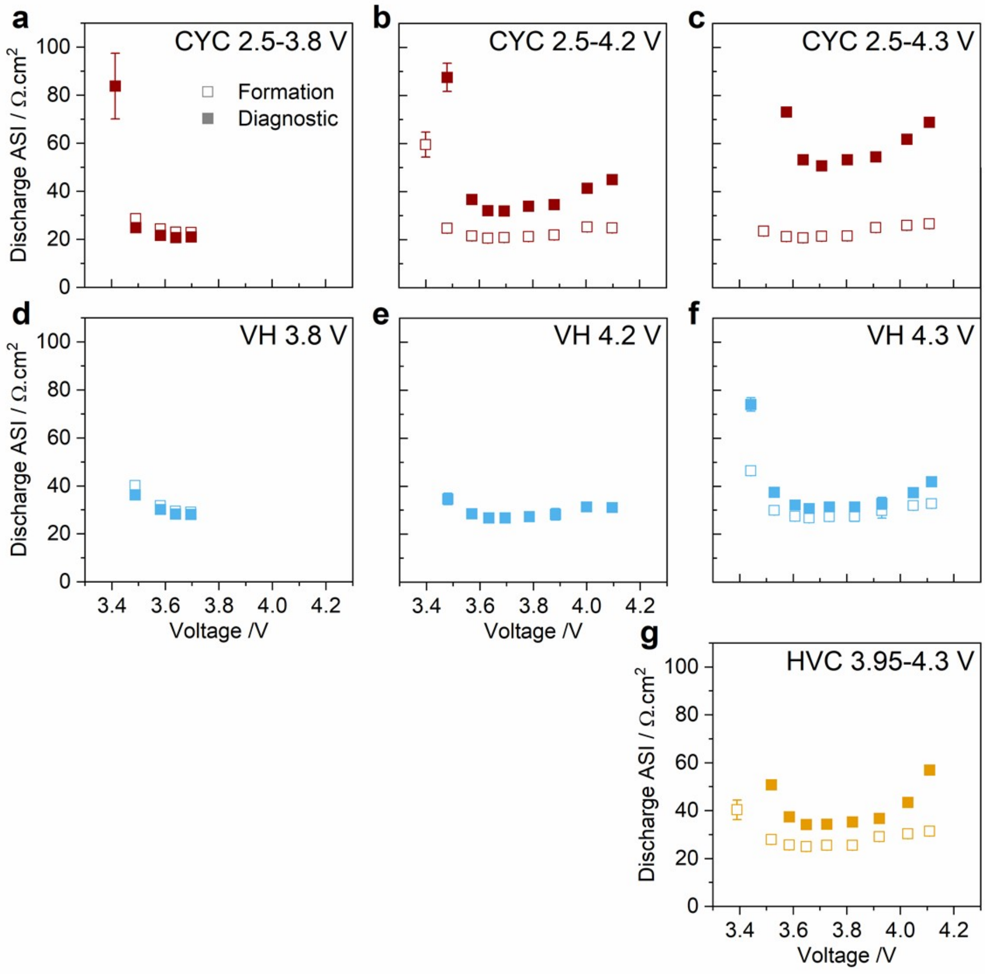

Figure S9. Full cell area specific impedance (ASI) as a function of cell voltage determined in hybrid pulse power characterization (HPPC) measurements after formation (open squares) and after aging, i.e. before diagnostic cycles, (filled squares). Aging by constant current-constant voltage cycling (CYC, a-c), voltage hold (VH, d-f), and high voltage cycling (HVC, g). Error bars on the plots represent the spread of two or three duplicate cells.

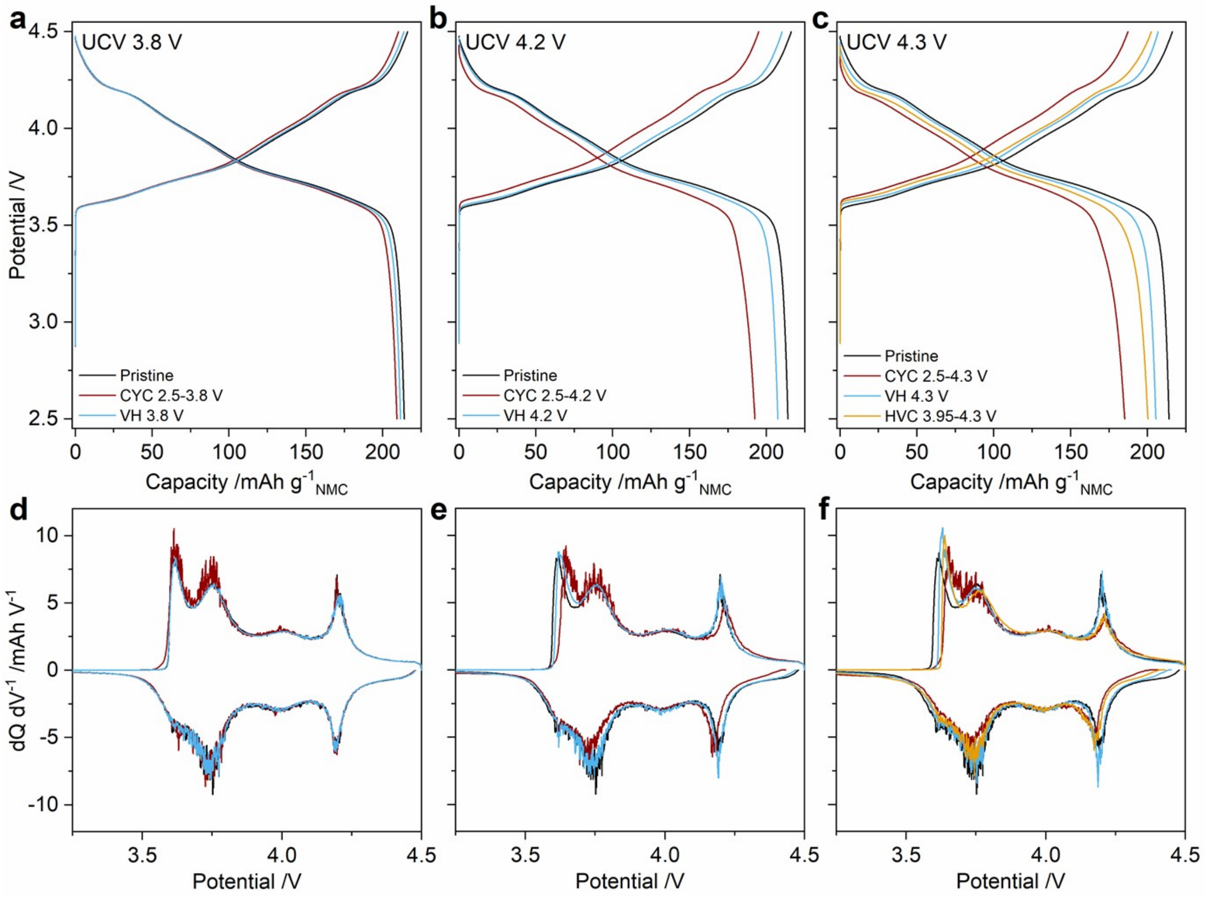

Figure S10. Representative NMC811 cathode (a-c) potential profiles and (d-f) differential voltage plots for Li/NMC811 half-cells with aged cathodes extracted from graphite/NMC811 full cells. Data is shown for the fifth C/20 cycle in the half-cell (3.0-4.5 V vs. Li/Li^+) and compared to the fifth C/20 cycle of a pristine (i.e. not aged in a full cell) NMC811 cathode cycled under the same conditions. Aging by constant current-constant voltage cycling (CYC), voltage hold (VH), and high voltage cycling (HVC). Data is grouped by full cell upper cutoff voltage, with 3.8 V in (a, d), 4.2 V in (b, e), and 4.3 V in (c, f).

Supplementary Note S3: EELS data processing

The spectrum images (SI) were processed using HyperSpy (a Python library for multidimensional data analysis). First, any unexpected spikes (cosmic rays, stray X-rays hitting the detector) in the data were removed. Then, the zero-loss peak position for each pixel of the SI was used to shift both low- and core-loss spectra so the center of the zero-loss peak was at 0 eV energy loss. A power law background subtraction was implemented from the core-loss spectra, by fitting the region before the onset of the oxygen K-edge. The Ni L edge was used to separate bulk and surface contributions to the spectrum image. Briefly, the Ni L edge was fitted at each pixel with: i) a power law background, ii) two Hartree-Slater generalized-oscillator-strength based ionization edges, iii) two Gaussian peaks (for the L_3 and L_2 white line peaks), and iv) their convolution with the low-loss peak to account for the sample thickness. Maps of Ni L_3 gaussian peak position were produced. The maps were then used to extract two binary masks for each spectrum image: surface (Ni L_3 peak position <855.2 eV) and bulk (Ni L_3 peak position >855.7 eV). Morphological operators were used as necessary to remove single pixels and fill in single-pixel gaps in the masks. The masks were used to average the raw data from the bulk and the surface, to give the data shown in Figure 6. Each of the averaged TM L edges (for all protocols, two particles for each, surface and bulk spectrum) were then fitted using the same approach as for the Ni edge fitting. This way, the positions of the Ni, Mn and Co L_3 peaks for the two regions were calculated (Figure 7).

TM L_3/L_2 edges correspond to transitions between 2p and unfilled 3d orbitals, which manifest as two peaks (L_3 and L_2) due to spin-orbit splitting. Several methods can be used to measure changes in the TM oxidation state from the L_3/L_2 edge.[4,5] In this work, we focus on the chemical shifts of the L_3 peak, as other methods (e.g. peak intensity ratio) require high signal-to-noise ratios, which need higher electron doses, potentially leading to sample damage. Reduction in the TM oxidation state causes the L_3 peak to shift towards lower electron energy

losses.[4,5] Moreover, we use the difference between the bulk and surface positions of the L_3 peak as a way of ensuring an internal reference.

On the other hand, the oxygen K edge consists of two main sets of peaks (individual peaks cannot be resolved with the energy resolution available, which is about 1 eV): (1) pre-edge peaks between 528 and 531 eV and (2) main peaks between 535 and 545 eV. The pre-edge peaks correspond to transitions between O 1s orbitals and the hybridized O 2p and TM 3d orbitals, while the main edge peaks are due to transitions from O 1s to hybridized O 2p and TM 4sp orbitals.[6,7] In layered cathode materials, such as NMCs, it has been shown that the pre-edge peaks become broader and shift towards higher energies (to >530 eV) with lower average oxidation state of the TMs (also demonstrated by a reference NiO spectrum, Figure S11a). This is due to the fact that the peak at ~528 eV corresponds to transitions from O 1s to hybridized Ni^{3+} or Ni^{4+} 3d-O 2p orbitals, while the peak at ~532 eV corresponds to transitions to the hybridized Ni^{2+} 3d-O 2p orbitals. [6–8]

The Ni L_3 position maps, used to calculate representative Mn and Co core-loss spectra, were also used to separate O K-edge into surface and bulk regions for each spectrum image. Each O K-edge spectrum was fitted using 5 Gaussian peaks, a single Hartree-Slater generalized oscillator strength based ionization edge and their convolution with the low-loss spectrum. The Gaussian peaks used were centered around the following energies: 528 and 531 eV for the pre-edge peaks, 540, 547 and 562 eV for the main edge peaks to best fit the data. Each Gaussian's center parameter was bound within ±0.5 eV for the pre-edge peaks and ±3 eV for the rest of the peaks.

To quantify the average reduction of the TMs in the surface and bulk spectra for all spectrum images, the difference between center of mass of the main edge peak CoM_m at about 540 eV and the pre-edge peaks CoM_p was calculated in terms of center parameters of the pre-edge peaks c_{p1}, c_{p2}, main peak c_m and their respective total areas A_{p1}, A_{p2} and A_m as:

$$\Delta CoM = CoM_m - CoM_p = \frac{c_m A_m}{A_m} - \frac{c_{p1}A_{p1} + c_{p2}A_{p2}}{A_{p1} + A_{p2}} \tag{S3}$$

Given the low S/N ratio and low energy resolution which prevents resolving each individual peak, this analysis is more robust than a full Gaussian deconvolution.

The results of this analysis for the TM L_3 edges and the O K edge are shown in Figure 7a in the main text. The error bars are twice the standard deviation error of the peak position parameter obtained during the fitting of a Gaussian function to the L_3 peak of each edge for the TM results and an equivalent obtained from error propagation for the center of mass difference for O-edge.

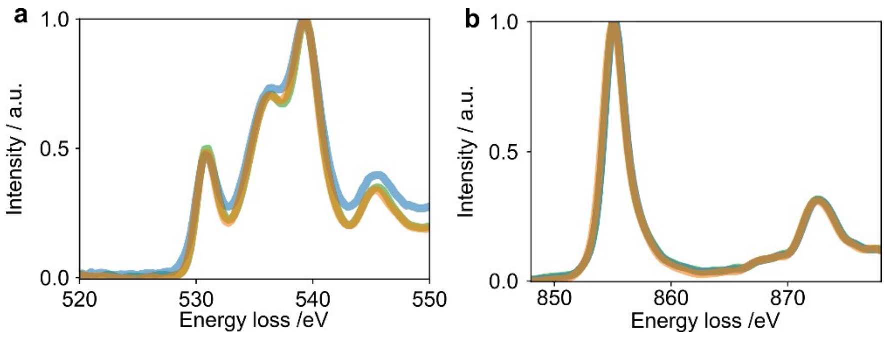

Figure S11. Electron energy loss spectra of NiO. (a) O K edge, (b) Ni L_3/L_2 edge. Each of the 3 spectra (blue, green, orange) is an average of all pixels of a spectrum image taken from three different particles in two microscope sessions. The data highlights the high accuracy and reproducibility of the EELS measurement of peak positions and acts as a reference for expected Ni^{2+} L_3 peak position: 855.13 ± 0.05 eV.

Supplementary Note S4: Capacity loss from impedance and the current rate dependence

Friedrich et al.[9] showed that after 1000 cycles in a pre-lithiated graphite/NMC811 cell cycled between 3.0-4.5 V vs Li/Li^+ at C/2 (cathode potential controlled versus a lithium metal reference electrode), that all the capacity loss attributed to impedance could be recovered in slow C/50 cycles. This is consistent with our own experiments in graphite/NMC811 cells (without pre-lithiation) after 500 cycles between 2.5-4.3 V at a C/2 rate (full cell voltage controlled, see Figure S12), which shows that the capacity at C/20, C/50, and C/100 are equivalent within ±2 mAh g^{-1}_{NMC}, but 20 mAh g^{-1}_{NMC} higher than that at C/2. In the analysis above, we quantified capacity "lost" due to impedance as the difference between the capacity on the constant-voltage charge step (C/40 cutoff current) in the formation and diagnostic cycles collected with constant-current at a C/20 rate.

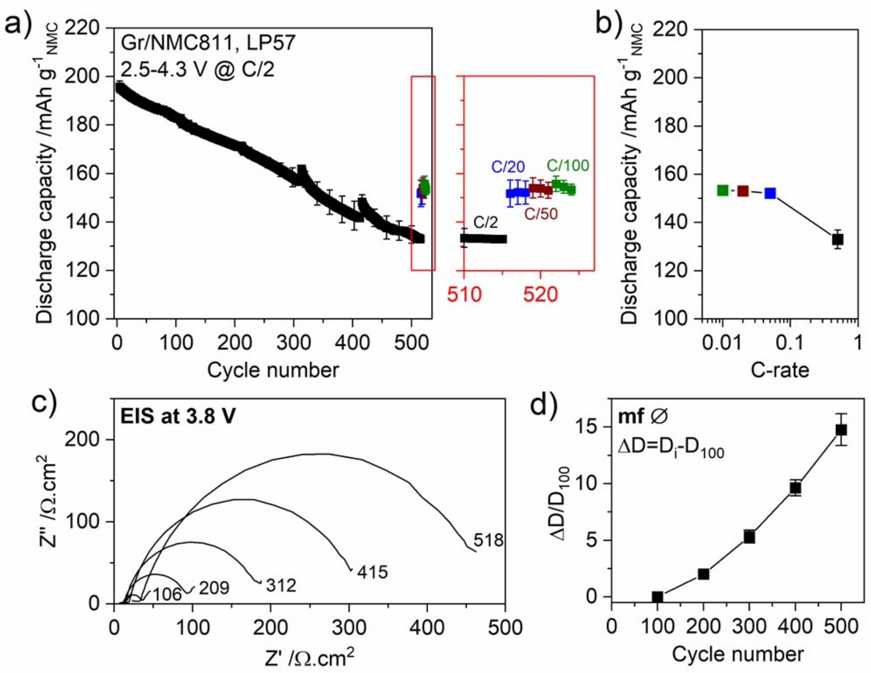

Figure S12. Discharge capacity and impedance rise of graphite/NMC811 full cells over 500 cycles. (a) Discharge capacity of graphite/NMC811 cells cycled between 2.5-4.3 V at a C/2 rate for 518 cycles followed by three C/20 cycles, three C/50 cycles, and three C/100 cycles. The C/2 cycles were conducted using a CCCV charging (C/20 terminal current in CV step) and CC discharging protocol. A magnified view of region indicated by the red rectangle is

shown on the right. (b) Discharge capacity versus C-rate in the final cycle collected at C/2, C/20, C/50, and C/100 in (a), which are cycles 515, 518, 521, and 524, respectively. In (a) and (b) data is shown for two duplicate cells with the error bars representing the spread. (c) EIS spectra collected every 103 cycles; spectra were measured at a full cell voltage of 3.8 V and at 25 °C. The cycle number is indicated next to the spectra. (d) The relative change in the diameter of the mid-frequency semicircle as a function of cycle number for the spectra shown in (c) as a function of cycle number.

Supplementary Note S5: Cracking from electrode manufacture and the impact on capacity loss and impedance rise

With a large number of NMC secondary particles already partially damaged or completely destroyed in calendered, pristine NMC electrodes (Figure 8), it raises the question – how important is particle cracking to the key issues of capacity loss and impedance rise? In the literature it appears that cracking is often investigated by focusing imaging efforts on particles that have (presumably) avoided damage from calendering (Type A).[10–14] However, since the same particle is not generally tracked before and after cycling this cannot be known for certain. Furthermore, important details of the electrode calendering and porosity are often omitted in the experimental details of literature reports.[10,14,15]

Prior literature has shown that NMC electrodes calendered to 30 % porosity show equivalent capacity to uncalendered electrodes (~50 % porosity) in the first cycle at a C/10 rate [16] – the NMC811 electrode in this work was calendered to 33 % porosity. Furthermore, for graphite/NMC811 full cells with calendered NMC electrodes, the impedance after three formation cycles is low (see Nyquist plot in Figure 4c), and only small differences are reported for EIS of uncalendered NMC electrodes versus those calendered to 30 % porosity.[16] Together, these findings indicate that mechanical cracking and/or pulverization of a significant portion

of NMC secondary particles does not *immediately* give rise to capacity loss or impedance rise. However, it is important to note that secondary particle cracking, induced by either electrode manufacture *or* the electrochemical protocol, may initiate degradation processes that manifest themselves in terms of capacity loss and impedance rise over the course of longer-term electrochemical aging.

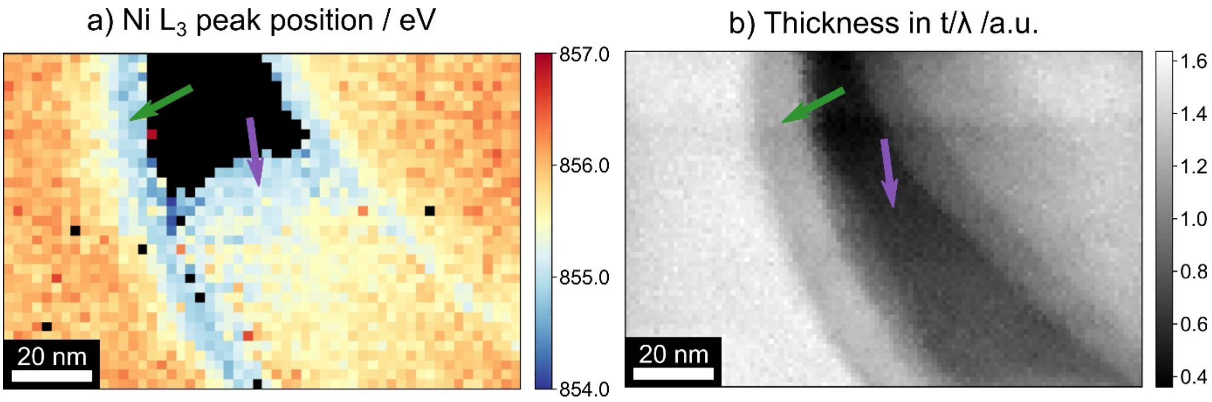

Figure S13. Ni L_3 peak position map (a) and thickness of the same region in t/λ (b) for a FIB lamella of the Formed sample, where λ is mean free path of electrons in the material, which is on the order of 100 nm. Thinner (along the electron beam direction, purple arrow) crystal shows a thicker apparent RSL compared to neighboring, thicker (along the beam direction, green arrow) crystals. Apparent curvature of crystal facets is due to sample drift during spectrum image acquisition. Abrupt change in t/λ at the edges of crystals is due to change in crystallographic phase and local density of the RSL compared to the bulk of the sample.

References

1 I. Bloom, A. N. Jansen, D. P. Abraham, J. Knuth, S. A. Jones, V. S. Battaglia and G. L. Henriksen, *J. Power Sources*, 2005, **139**, 295–303.

2 I. Bloom, J. Christophersen and K. Gering, *J. Power Sources*, 2005, **139**, 304–313.

3 I. Bloom, L. K. Walker, J. K. Basco, D. P. Abraham, J. P. Christophersen and C. D. Ho, *J. Power Sources*, 2010, **195**, 877–882.

4 R. F. Egerton, *Electron Energy-Loss Spectroscopy in the Electron Microscope*, Springer Science and Business Media LLC, 3rd edn., 2011.

5 H. Tan, J. Verbeeck, A. Abakumov and G. Van Tendeloo, *Ultramicroscopy*, , DOI:10.1016/j.ultramic.2012.03.002.

6 W. S. Yoon, M. Balasubramanian, K. Y. Chung, X. Q. Yang, J. McBreen, C. P. Grey and D. A. Fischer, *J. Am. Chem. Soc.*, 2005, **127**, 17479–17487.

7 W.-S. Yoon, K. Y. Chung, J. McBreen, D. A. Fischer and X.-Q. Yang, *J. Power Sources*, 2007, **174**, 1015–1020.

8 S. Hwang, W. Chang, S. M. Kim, D. Su, D. H. Kim, J. Y. Lee, K. Y. Chung and E. A. Stach, *Chem. Mater.*, 2014, **26**, 1084–1092.

9 S. K. Heiskanen, N. Laszczynski and B. L. Lucht, *J. Electrochem. Soc.*, 2020, **167**, 100519.

10 H. H. Ryu, K. J. Park, C. S. Yoon and Y. K. Sun, *Chem. Mater.*, 2018, **30**, 1155–1163.

11 A. O. Kondrakov, A. Schmidt, J. Xu, H. Geßwein, R. Mönig, P. Hartmann, H. Sommer, T. Brezesinski and J. Janek, *J. Phys. Chem. C*, 2017, **121**, 3286–3294.

12 S. Schweidler, L. De Biasi, G. Garcia, A. Mazilkin, P. Hartmann, T. Brezesinski and J. Janek, *ACS Appl. Energy Mater.*, 2019, **2**, 7375–7384.

13 H. Liu, M. Wolf, K. Karki, Y. S. Yu, E. A. Stach, J. Cabana, K. W. Chapman and P. J. Chupas, *Nano Lett.*, 2017, **17**, 3452–3457.

14 E. J. Lee, Z. Chen, H. J. Noh, S. C. Nam, S. Kang, D. H. Kim, K. Amine and Y. K. Sun, *Nano Lett.*, 2014, **14**, 4873–4880.

15 K. Ishidzu, Y. Oka and T. Nakamura, *Solid State Ionics*, 2016, **288**, 176–179.

16 H. Zheng, L. Tan, G. Liu, X. Song and V. S. Battaglia, *J. Power Sources*, 2012, **208**, 52–57.
